# Effect of Different Culture Conditions on Gene Expression Associated With Cyst Production in Populations of *Artemia franciscana*

**DOI:** 10.3389/fgene.2022.768391

**Published:** 2022-03-31

**Authors:** Margarita Parraguez

**Affiliations:** Laboratorio de Genética, Acuicultura y Biodiversidad, Departamento de Ciencias Biológicas y Biodiversidad, Universidad de Los Lagos, Osorno, Chile

**Keywords:** *Artemia franciscana*, diapause cyst, chorion thickness, differential expression, semiquantitative RT-PCR

## Abstract

*Artemia franciscana* inhabits hypersaline environments in the Americas and has a well-adapted reproductive system that allows it to survive in these extreme conditions, represented by the production of diapause cysts (oviparous reproduction). This reproduction mode is controlled by numerous genes that are expressed in response to different environmental stressors, enabling this species to avoid population extinction. However, to date, the expression of these genes has not been sufficiently studied to clarify their levels in response to a combination of different environmental factors under controlled conditions. We analyzed the expression of eight genes related to oviparous reproduction (*SGEG*, *Arp-CBP*, *artemin*, *BRCA1*, *p8*, *ArHsp21*, *ArHsp22*, and *p26*) to determine their association with cyst production in two populations of *A. franciscana* with contrasting phenotypes, one with high (Barro Negro, BNE, Chile) and one with low (San Francisco Bay, SFB, United States) cyst production. Populations were cultured under controlled conditions of salinity (SAL, 35 and 75 ppt), photoperiod (PHO, 12L:12D and 24L:00D), iron concentration (IC, 0[Fe] and 5[Fe]), and microalgae diet (DIE; *Dunaliella tertiolecta* (DUN) and *Tetraselmis suecica* (TETRA)). Sixteen treatments were performed by combining the two conditions of each of the four factors. Data on nine reproductive parameters per female were recorded, including the percent of offspring encysted (%) (POE). The gene expression levels were analyzed by semiquantitative RT-PCR. The mean POE was significantly greater in BNE than in SFB (32.40 versus 12.74%, Mann–Whitney’s test, *p* < 0.05). Significantly upregulated expression of seven genes in BNE (more than twofold, *p* < 0.05) was observed in 38.28% of the treatments (e.g., DUN-75ppt-12L:12D-5[Fe] and TETRA-35ppt-12L:12D-5[Fe]). In SFB, seven genes showed significant differential expression, but most were downregulated in 29.69% of the treatments (e.g., DUN-75ppt-12L:12D-0[Fe] and DUN-75ppt-24L:00D-0[Fe]). Multiple regression analyses indicated that in BNE, five genes (*SGEG*, *artemin*, *Arp-CBP*, *p8*, and *BRCA1*) and three environmental factors (DIE, SAL, and IC) were important predictor variables for the POE response variable given that all of them were included in the highest-ranking models. In SFB, only two genes (*ArHsp21* and *artemin*) and one environmental factor (SAL) were important explanatory variables in the highest-ranking models. It was concluded that the BNE population presented a characteristic gene expression pattern that differed from that of the SFB population. This pattern might be related to the marked oviparous reproduction of the BNE population. This gene expression pattern could be useful for monitoring the reproductive mode leading to diapause in *Artemia* and to assist with intensive cyst production in pond systems.

## Introduction

*Artemia* is a branchiopod crustacean that inhabits hypersaline environments on different continents (Sorgeloos et al., 1986). These environments constitute extremes, given that they are subject to strong ultraviolet radiation, high salinity, high-temperature variation, desiccation and hydration cycles, oxidation, and anoxia (Tanguay et al., 2004; MacRae 2016). From a reproductive point of view, this organism is characterized by two reproductive modes, oviparous and ovoviviparous, enabling it to adapt to these extreme environments. The first breeding mode is a diapause process driven by environmental factors related to variations in salinity, temperature, photoperiod, iron concentration, and diet (Versichele and Sorgeloos, 1980; Wang et al., 2017, Wang et al., 2019). The result is the production of a cyst that contains a gastrulae embryo in the diapause stage and is coated with a rigid chitinous shell (Jackson and Clegg, 1996; Liang and MacRae, 1999; MacRae 2003). This structure corresponds to the chorion and protects the embryo from mechanical damage and physicochemical factors (Clegg and Conte, 1980; Tanguay et al., 2004; MacRae 2016). The oviparous reproductive mode is a strategy that enables *Artemia* to produce offspring during adverse environmental conditions, thus increasing the survival probabilities of the population in future generations (Browne 1980).

*Artemia franciscana* is a species of the New World and presents either oviparous or ovoviviparous reproductive modes. In natural populations, the oviparous reproduction rate can vary widely. For example, in North American populations, it has been observed that the average percent of cysts per female can fluctuate between 18.4% and 29.3%, considering a salinity of 90 ppt (Browne et al., 1984). A similar pattern of variation has been observed in Chilean populations of this species, with an average number of between 57 and 324 total cysts per female (Gajardo et al., 1998), or an average percent of 27%–51% cysts per female in natural populations maintained under laboratory conditions at 65 ppt salinity (Gajardo et al., 2001). This variation in cyst production has been related to the effect of environmental physicochemical factors. In fact, in extensive *Artemia* cultures undertaken in other countries, it has been observed that cyst production varies according to the duration of the dry season, environmental temperature, occasional rainfall that modifies the ionic concentration of the water, and fluctuations in microalgae abundance, among other factors (Van Stappen et al., 2020). To date, in Chile, systematic studies focused on the association of cyst production and physicochemical environmental factors are scarce. One experimental study on this topic performed in Chile referred to the effect of temperature and salinity on cyst production in a population of *Artemia* of North American origin cultured in northern Chile (Moraga et al., 2015). This analysis shows that the variables temperature (25°C) and salinity (120 g/L), independently or combined, can increase cyst production.

Studies on factors influencing the diapause reproductive mode in *Artemia* began in the 1960s. These initial studies identified oxygen concentration (Dutrieu 1960) and salinity as important factors that could increase cyst production (Versichele and Sorgeloos, 1980). Other studies indicated that low salinities (<60 ppt) would be important to increase cyst production (Berthélémy-Okazaki and Hedgecock, 1987), although more recent studies have shown contradictory results (Castro-Mejía et al., 2013; Wang et al., 2017). Similarly, it has also been observed that increased iron concentration (10–30 mg/L) would correlate positively with cyst production per female (22%–71%) under a system of continuous aeration. This effect would be even more apparent when oxygen concentration is decreased (Versichele and Sorgeloos, 1980). Given that the formation of the cyst chorion depends on hematin secretion, which, in turn, depends on the presence of iron in the environment (Liu et al., 2009), increased cyst production would concur with a greater presence of this ion in the environment. On the other hand, studies referring to the use of different diets indicate that feed has a differential effect on cyst production in *Artemia* (Vartak and Joshi, 2002; Esteve and ElMasri, 2007). In fact, the studies of Vartak and Joshi (2002), who used 12 diets that included three different microalgae (*Chaetoceros*, *Spirulina*, and *Tetraselmis*), revealed significant differences in cyst production per female, with the latter two microalgae producing the highest yield. Photoperiod is another factor that could also influence cyst production, although with a minor effect (Sorgeloos et al., 1976). In this respect, it has been observed that a short day tends to produce an increase in cyst production (Nambu et al., 2004; Wang et al., 2017). However, the incidence of this factor would also be related to temperature variation (19°C–28°C).

The genetic-molecular basis of the diapause is a topic of general interest that has been approached in some species of invertebrates over the past years (Chen et al., 2017; Pruisscher et al., 2018; Ren et al., 2018). These studies have revealed various genes that would control the existence of this reproductive process, such as *period*, *kinesin*, *carnitine O-acetyltransferase*, and *timeless* in butterflies *Pararge aegeria* (Pruisscher et al., 2018) and the circadian clock gene in the diptera *Delia antiqua* (Ren et al., 2018). Similarly, transcriptomic studies on the silkworm *Bombyx mori* have revealed the existence of approximately 180 differentially expressed genes, with a high expression level related to the diapause process (Chen et al., 2017). The first genetic studies carried out in *Artemia* suggest a genetic and environmental component to the determination of reproductive mode in this crustacean (Browne et al., 1984). Studies estimating genetic-quantitative parameters support an important genetic determination of reproductive traits in *Artemia*, such as a number of offspring at first brood, given the existence of a significant genetic component controlling this trait (h^2^ = 0.34) (Shirdhankar et al., 2004). Progress made in recent years through gene expression analysis has identified various genes that could be associated with diapause in *Artemia*, such as *SGEG*, *Arp-CBP*, *BRCA1*, *p8*, *artemin*, *ArHsp21*, *ArHsp22*, and *p26* (Qiu et al., 2007; Qiu and MacRae, 2008a, Qiu and MacRae, 2008b; Liu et al., 2009; Dai et al., 2011; King et al., 2013; King et al., 2014; Zhou et al., 2014; Ye et al., 2017) (Table 1). These genes participate at different levels, in the formation of the cyst chorion (e.g., *SGEG* and *Arp-CBP*), in the control of cellular division (e.g., *BRCA1* and *p*8), or as molecular chaperones whose function is to avoid denaturation of the proteins (e.g., *artemin*, *ArHsp21*, *ArHsp22*, and *p26*). For example, Liu et al. (2009) described *SGEG* gene that participates in chorion formation; inactivation of this gene produces cysts that lose their capacity to resist different environmental stresses, such as UV light, desiccation, and high temperatures. This gene encodes two peptides (*SGEG*1 and *SGEG*2) secreted by the shell gland, which conform to the outer and inner layers of the chorion (Dai et al., 2011). In the case of the genes related to cellular division, Qiu and MacRae (2007) described *p8* gene, which presents increased expression one day postfertilization in diapause-destined embryos. This class of genes has also been associated with diapause in *A. franciscana* and is often upregulated, in this species (*BRCA1* and *p8*) (Qiu et al., 2007). Additionally, the genes associated with environmental responses, such as *artemin*, *ArHsp21*, *ArHsp22*, and *p26*, have been related to environmental factors such as temperature and freezing–desiccation cycles (King et al., 2013), as well as with photoperiod, as occurs with genes that encode *Arp-CBP* (Wen-Ming et al., 2013).

**TABLE 1:** Genes related to diapause in *Artemia* according to literature.

Gene names	Gene codes	Function	References
**Formation of the cyst chorion**			
Shell gland-specific gene I	*SGEG*	Involved in cyst shell formation, protecting the encysted embryos from environmental stress. *SGEG* begins to accumulate a day before ovulation and decreases 2 days later	Liu et al. (2009), Dai et al. (2011)
Chitin-binding protein	*Arp-CBP*	Chitin-binding protein contributes to the formation of the cyst coat, which is essential for embryo survival	Qiu et al. (2007)
**Control of cellular division**			
Ubiquitin	*BRCA1*	They may influence cell growth and division during the transition to diapause	Qiu et al. (2007)
*p8*	*p8*	Stress-associated transcription cofactor known to modulate cell growth, division, and apoptosis. It may modulate gene expression during diapause in *Artemia* embryos	Qiu et al. (2007), King and MacRae (2012)
**Related to environmental responses**			
artemin	*artemin*	*Artemin* is an iron storage protein that is structurally similar to ferritin. This protein has high thermal stability and functions as a chaperone. Therefore, *artemin* protects cysts from desiccation and freezing	Chen et al. (2007)
Small heat shock protein	*ArHsp21*	*ArHsp21* has the capacity to protect cell proteins during the metabolic decline associated with diapause. It may prevent irreversible protein denaturation when diapause embryos encounter harsh environments. This protein potentially contributes to apoptosis inhibition. Plays a minor role in diapause formation	Qiu and MacRae (2008a)
Small heat shock protein	*ArHsp22*	It is the only heat-induced sHSP in adults, and it prevents irreversible protein denaturation	Qiu and MacRae (2008b)
Small heat shock proteinChaperon	*p26*	It is a molecular chaperone, which reversibly translocates to nuclei during stress and confers thermotolerance, preventing irreversible protein denaturation. It expresses in *Artemia franciscana* embryos 2 days after fertilization	Qiu et al. (2007)

Available studies on molecular genetics have allowed the identification of various genes related to the reproductive mode leading to diapause in *Artemia.* However, their usefulness as genetic markers to predict or assess cyst production in controlled cultures of this crustacean has not yet been explored. The aim of this study was to determine the association between previously described gene markers of diapause and cyst production in two populations of *Artemia*. To achieve this, contrasting phenotypes for this trait (high and low cyst production), were analyzed. We analyzed eight genes through gene expression analysis by semiquantitative RT-PCR, a simple and low-cost technique to scale at the production level (Capuano et al., 2013), in order to determine which of them are associated with the oviparous reproductive mode. Given that these genes respond differently to different environmental factors, in this study, we analyzed gene expression considering four physicochemical and nutritional factors, including diet, salinity, iron concentration, and photoperiod. This information may be useful for early diagnosis of the existence of populations or strains with a high cyst production capacity.

## Materials and Methods

### Origin of Populations Studied

Two populations of *Artemia* were analyzed, corresponding to Barro Negro (BNE) and San Francisco Bay (SFB). The population of BNE originates from the Salar de Atacama, Chile (23°17′S 68°10′W), and was transported live to the laboratory in 2011 for conditioning in aquaria with 20-L capacity in artificial seawater at 35 ppt. This population is characterized as producing a high average percentage of cysts (42%) in comparison to other Chilean populations, such as Tebenquiche (3.28%) and Lo Valdivia (0.0%) (unpublished data). The SFB population corresponds to the species *A. franciscana* (37°39′N 122°25′W) (code 1364, *Artemia* Reference Center, Ghent University, Belgium) and was included as a reference population for this study. Previous records obtained in our laboratory indicate that this population can produce an average cyst percentage of 27% (Gajardo et al., 2001). Given these reproductive characteristics, the BNE population was considered a high cyst-producing population, while the SFB population corresponded to a low cyst-producing population.

### Breeding Stock and Culture Conditions

The breeding stocks of both populations were formed in 2016, based on larvae produced by adult specimens (*n* = 50 pairs of each population) that were available in the laboratory, cultured at 75 ppt. Offspring at the larval stage of each population was cultured in two different aquaria containing 1 L of seawater at 75 ppt. One aquarium was fed with *Dunaliella tertiolecta* (DUN) and the other with *Tetraselmis suecica* (TETRA). Specimens were maintained until they reached sexual dimorphism. These breeders were used to conform to experimental paired crosses.

### Establishment of Experimental Pairs and Experimental Design

We performed between 5 and 32 experimental paired crosses, depending on the treatment, using individual glass beakers containing 100 ml of seawater at a temperature of between 23°C and 27°C. The crosses were undertaken using a multifactorial design with two different conditions for each factor: 1) diet (*D. tertiolecta* and *T. suecica*), 2) salinity (35 and 75 ppt), 3) photoperiod (12Light:12Dark and 24Light:00Dark), and 4) iron concentration (0 mg/L of [Fe^2+^] and 5 mg/L of [Fe^2+^]). No artificial aeration was used. These microalgae were administered at a concentration of 1 × 10^6^ cell/beaker/day (Gajardo et al., 2001). This experimental design resulted in a total of 16 treatments, by combining the two conditions of each of the four factors (Table 2).

**TABLE 2:** Codes used for treatments in this work.

No. of treatment	Diet	Salinity (ppt)	Photoperiod	Iron concentration	Code
1	*Dunaliella tertiolecta*	35	12Light:12Dark	[0 mg Fe^2+^/L]	DUN-35ppt-12L:12D-0[Fe]
2	*D. tertiolecta*	35	12Light:12Dark	[5 mg Fe^2+^/L]	DUN-35ppt-12L:12D-5[Fe]
3	*D. tertiolecta*	35	24Light:00Dark	[0 mg Fe^2+^/L]	DUN-35ppt-24L:00D-0[Fe]
4	*D. tertiolecta*	35	24Light:00Dark	[5 mg Fe^2+^/L]	DUN-35ppt-24L:00D-5[Fe]
5	*D. tertiolecta*	75	12Light:12Dark	[0 mg Fe^2+^/L]	DUN-75ppt-12L:12D-0[Fe]
6	*D. tertiolecta*	75	12Light:12Dark	[5 mg Fe^2+^/L]	DUN-75ppt-12L:12D-5[Fe]
7	*D. tertiolecta*	75	24Light:00Dark	[0 mg Fe^2+^/L]	DUN-75ppt-24L:00D-0[Fe]
8	*D. tertiolecta*	75	24Light:00Dark	[5 mg Fe^2+^/L]	DUN-75ppt-24L:00D-5[Fe]
9	*Tetraselmis suecica*	35	12Light:12Dark	[0 mg Fe^2+^/L]	TETRA-35ppt-12L:12D-0[Fe]
10	*T. suecica*	35	12Light:12Dark	[5 mg Fe^2+^/L]	TETRA-35ppt-12L:12D-5[Fe]
11	*T. suecica*	35	24Light:00Dark	[0 mg Fe^2+^/L]	TETRA-35ppt-24L:00D-0[Fe]
12	*T. suecica*	35	24Light:00Dark	[5 mg Fe^2+^/L]	TETRA-35ppt-24L:00D-5[Fe]
13	*T. suecica*	75	12Light:12Dark	[0 mg Fe^2+^/L]	TETRA-75ppt-12L:12D-0[Fe]
14	*T. suecica*	75	12Light:12Dark	[5 mg Fe^2+^/L]	TETRA-75ppt-12L:12D-5[Fe]
15	*T. suecica*	75	24Light:00Dark	[0 mg Fe^2+^/L]	TETRA-75ppt-24L:00D-0[Fe]
16	*T. suecica*	75	24Light:00Dark	[5 mg Fe^2+^/L]	TETRA-75ppt-24L:00D-5[Fe]

### Record of Breeding Parameters

We kept a daily record of seven female reproductive characters comprising the following: 1) Reproductive period (days), 2) Total offspring (number), 3) Percent of offspring as nauplii (larvae) (%), 4) Percent of offspring encysted (%) (POE), 5) Percent of offspring encysted at first brood (%) (POE-1), 6) Percent of offspring encysted at second brood (%) (POE-2), and 7) Percent of offspring encysted at third brood (%) (POE-3). Total offspring, whether larvae or cysts, was determined by counting them under a stereoscopic microscope. Only the brown or dark brown cysts were counted since they are considered viable cysts, according to the literature on available color code, as a rough estimate of the hemoglobin concentration in *Artemia* (Vos et al., 1984; Sorgeloos et al., 1986). The POE-3 parameter was evaluated because, at this stage, it is possible to define clearly the oviparous reproductive mode expressed by each female of *Artemia* (Berthélémy-Okazaki and Hedgecock, 1987). In the event of female death during the first 3 days of the experiment, a replacement female was used to continue the experiment. Otherwise, the flask was removed to avoid the desynchronization of the experiment between the different flasks. At the end of the experiment (i.e., third brood), we stored the intact adult females in RNA-Latter at −20°C for subsequent gene expression analysis. In addition, we determined the diameter of hydrated (DHC) or decapsulated (DDC) cysts (µm), together with chorion thickness (CT). This parameter was analyzed due to it varying as a function of salinity, iron concentration, and diet availability (Wang et al., 2019), and therefore, its analysis may have an applied value. Decapsulation of the cysts was performed according to the Sorgeloos et al. (1986) protocol. The brown or dark brown cysts were photographed in a stereoscopic microscope (ZEISS Axiocam 208 color), equipped with Labscope release version 2.9 (36×) image software, under incident illumination. We determined cyst diameter using ImageJ analysis software, with object measurement function. We obtained CT by calculating the difference between the DHC minus the DDC, divided by two (CT = (DHC − DDC)/2), according to Peykaran Mana et al. (2011).

### Ribonucleic Acid Extraction and cDNA Synthesis

Adult females with oocyte-filled lateral pouches were used to extract total RNA, using TRIZOL (Chomczynski 1993). Total RNA was then resuspended in 30 µl of sterilized water treated with diethyl pyrocarbonate (DEPC). We performed quantification of total RNA in a spectrometer at 260/280 nm. To avoid RNA degradation, it was stored overnight at −20°C prior to its use for cDNA synthesis. We carried out cDNA synthesis using the First-Strand cDNA Synthesis kit, according to the manufacturer’s instructions (Thermo Scientific, Waltham, MA, USA, k1612), using 1 µg of RNA and first oligo (dT)_18_. We then stored the cDNA samples at −20°C prior to analysis.

### Semiquantitative Real-Time PCR Analysis

We carried out the PCR in a final volume of 10 μl containing 2 μl of cDNA, 2 µl of reaction buffer C (1×), 0.93 µl of MgCl_2_ (2.3 mM), 0.2 µl of each dNTP (0.2 mM), 0.3 µl (0.3 μM) of forward and reverse primers, and 0.15 µl of Taq polymerase (Kapa Biosystem Inc., Wilmington, MA, USA) (0.075 U/µl). We used the following thermal profile: denaturalization at 94°C for 5 min followed by 26 cycles at temperatures of 94°C for 45 s, 56°C for 45 s, 72°C for 45 min, and a final extension at 72°C for 5 min. The number of cycles used was identified by means of a gradient of amplification cycles that varied between 18 and 34 cycles, which involved a comparative analysis of the intensity of the bands of the amplicons in agarose gels that were obtained at different cycle numbers. PCR products were visualized in Metaphor agarose gel at 2.5% (Lonza Walkersville, Inc., Walkersville, MD, USA) for visualization under UV light prior to staining with SYBR green (Invitrogen, Carlsbad, CA, USA, S7563). We photographed the gels with a 10 mpx digital camera for subsequent analysis of the bands, using a manual mode setting. We analyzed the following genes: *SGEG*, *Arp-CBP*, *BRCA1*, *p8*, *artemin*, *ArHsp21*, *ArHsp22*, *p26*, and *β-actin*. The latter was included as a housekeeping gene. We chose *p8*, *BRCA1*, *p26*, *Arp-CBP*, *artemin*, *ArHsp21*, and *ArHsp22* genes, because according to Qiu et al. (2007), these genes present a significant overexpression in *A. franciscana*. Moreover, Clegg and Gajardo (2009) described *artemin* and *p26* proteins in cysts of Chilean *Artemia* populations, in substantial although variable amounts. Because *SGEG* gene has not been described in *A. franciscana*, the PCR products of the two populations studied were sequenced (GenBank accession numbers OM525818–OM525823) in order to establish their level of identity with *SGEG* gene described *A. parthenogenetic* (GenBank accession number EU683079). This analysis showed a high level of identity between both sequences (nucleotide = 93.5%, protein = 84.4%), which supports that it is the same gene. We obtained the primers from literature, or alternatively, they were designed for this study using the Geneious program (version 11.1.5), based on the published sequences of each gene in the GenBank (Supplementary Table S1).

### Gene Expression Analysis

We used a semiquantitative RT-PCR protocol to perform gene expression analysis that included estimation of amplified product quantity at the end of the reaction, through quantification of band intensity in agarose gels (Overturf, 2009). We determined the gene expression level of a particular gene by quantification of band intensity present in agarose gels with respect to *β-actin* gene expression. To do so, we obtained a photograph of the gel and proceeded with quantification of bands intensity using the ImageJ program (Version 1.52) through the Integrated Density function. The level of gene expression, either up or down, was normalized by means of the candidate gene/*β-actin* gene ratio. The ratio obtained was transformed on a base-two logarithm scale (Log_2_), where Log_2_ values greater than two were considered significant as up expression and values less than 0.5 as down expression (Smyth et al., 2003). Normalized gene expression levels were visualized in a heatmap through the Z-score rank, using the Heatmapper web server available at http://www.heatmapper.ca/ (Babicki et al., 2016).

### Statistical Analysis

We determined the normality of the reproductive variables using Shapiro’s test, while homogeneity of variance was evaluated with Levene’s test (*p* < 0.05). When the variables did not meet the normality criterion, we used the Mann–Whitney nonparametric test (*p* < 0.05) to establish the significance of differences between factors. We also performed the Kruskal–Wallis nonparametric test (*p* < 0.05) to evaluate the significance of difference among all treatments. When the differences were significant, we used Dunn’s post-hoc multiple-comparisons test with Bonferroni adjustment (Rice 1989; Sokal and Rohlf, 1995).

Forward selection stepwise multiple regression analyses were performed on POE with the expression level of each gene (*SGEG*, *Arp-CBP*, *BRCA1*, *p8*, *artemin*, *ArHsp21*, *ArHsp22*, and *p26*) and the environmental factors (DIE, SAL, IC, and PHO) variables, as potential predictors to identify which variable, or combinations of variables, was the most highly correlated with, and therefore possibly causally linked to, POE. This analysis was performed using the statistical package MuMIn version 1.43.17 designed for multi-model inference (Bartoń 2013). We used Akaike’s information criterion adjusted for small sample size ( $AIC_{c}$ ) (Burnham and Anderson, 2002) to determine model support. We considered models with substantial empirical support (plausible models) when delta AICc values were less than 2. All the statistical analyses were carried out with the RStudio program (version 4.1.1).

## Results

### Reproductive Analysis

**FIGURE 1:**
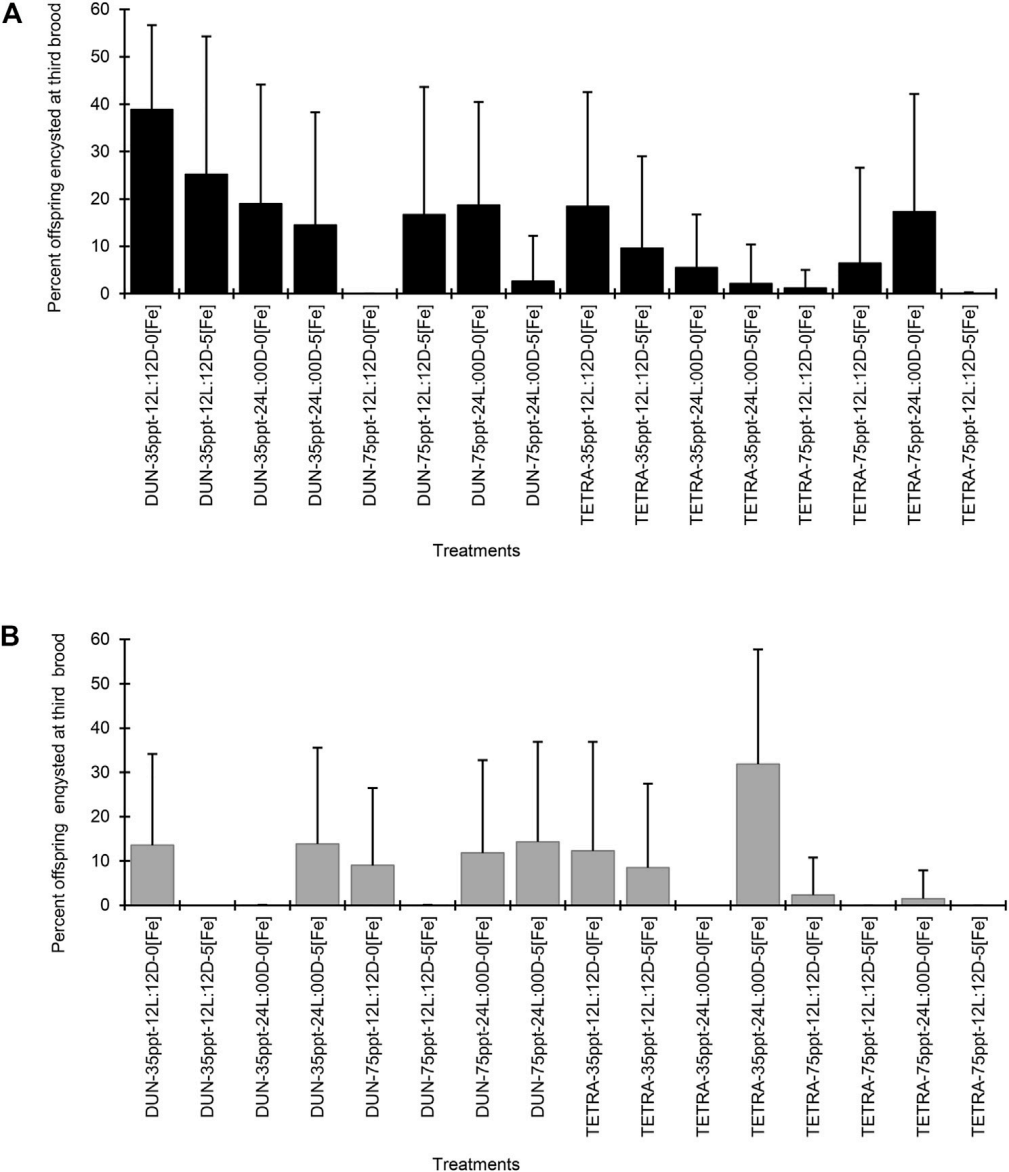
Percent offspring encysted at third brood in two populations of *Artemia franciscana* exposed to a combination of different diets, salinities, photoperiods, and iron concentrations treatments. **(A)** Barro Negro population and **(B)** San Francisco Bay population. The error bars indicate one SD.

In the BNE population, the timing of the third brood varied between 8.23 and 14.43 days (Table 3). However, between treatments, this variation was not significant in the majority of cases (Dunn’s test, *p* < 0.05), except between treatments DUN-35ppt-12L:12D-5[Fe] and DUN-35ppt-12L:12D-0[Fe] (8.23 and 13.06 days, respectively). Total offspring varied greatly, between 196.10 and 448.45; however, these were only statistically significant between the TETRA-35ppt-12L:12D-0[Fe] and DUN-35ppt-24L:00D-0[Fe] (Dunn’s test, *p* < 0.05) treatments. The differences in POE between treatments were highly significant (Kruskal–Wallis’s test, *p* < 0.0001) (min = 0.08, max = 82.17). Furthermore, 14/120 pairwise comparisons were significant (Dunn’s test, *p* < 0.05), for example, between DIE treatments DUN-75ppt-12L:12D-0[Fe] and DUN-35ppt-12L:12D-0[Fe] (2.46 and 82.17%, respectively), as well as between DIE treatments TETRA-75ppt-24L:00D-5[Fe] and TETRA-75ppt-24L:00D-0[Fe] (0.08% and 48.28%, respectively). The same pattern of significant variation in POE between treatments was also observed in POE-3 (Kruskal–Wallis’s test, *p* < 0.0001) (min = 0.00%, max = 38.89%) and in 9/120 pairwise comparisons (Dunn’s test, *p* < 0.05) (Figure 1A). In contrast, in the case of SFB, although there was variation in POE between treatments (min = 0.00%, max = 32.77%) and in POE-3 (min = 0.00%, max = 31.87%) (Table 4; Figure 1B), statistical analysis did not show statistically significant differences in the pairwise comparisons, except in two comparisons at POE-3 (Dunn’s test, *p* < 0.05). Similarly, considering the overall data (i.e., mean of all treatments), POE was significantly greater in BNE than in SFB (32.40 versus 12.74%, Mann–Whitney’s test, *p* < 0.05). The higher cyst production of BNE compared to SFB was also observed in the other reproductive parameters analyzed. When we consider the number of oviparous females recorded in each population with respect to total females analyzed (Tables 3, 4), BNE presented a greater proportion of females (54.89%, 129/235) of this class, compared to SFB (28.04%, 53/189).

**TABLE 3:** Summary of reproductive parameters of 16 treatments recorded for Barro Negro population.

Treatments	Total/oviparous females	Reproductive period at the 3rd brood (days)	Total offspring (number)	Offspring (%)					
				Nauplii	Encysted	Encysted 1st brood	Encysted 2nd brood	Encysted 3rd brood	
		$\bar{x}\left(\pm \text{SD}\right)$	$\bar{x}\left(\pm \text{SD}\right)$	$\bar{x}\left(\pm \text{SD}\right)$	$\bar{x}\left(\pm \text{SD}\right)$	$\bar{x}\left(\pm \text{SD}\right)$	$\bar{x}\left(\pm \text{SD}\right)$	$\bar{x}\left(\pm \text{SD}\right)$	
DUN-35ppt-12L:12D-0[Fe]	32/32	13.06^a^ (2.93)	299.31^ab^ (115.36)	17.83^a^ (21.36)	82.17^a^ (21.36)	10.38^a^ (12.24)	32.90^a^ (19.93)	38.89^a^ (17.79)	
DUN-35ppt-12L:12D-5[Fe]	13/7	8.23^b^ (2.24)	301.31^abc^ (134.24)	65.84^bc^ (39.47)	34.16^bc^ (39.47)	2.09^ab^ (4.91)	6.86^ab^ (12.43)	25.21^ab^ (29.12)	
DUN-35ppt-24L:00D-0[Fe]	11/10	11.45^ab^ (6.14)	448.45^a^ (147.42)	52.96^abc^ (38.10)	47.04^abc^ (38.10)	9.59^ab^ (11.13)	18.47^ab^ (18.34)	18.99^ab^ (25.16)	
DUN-35ppt-24L:00D-5[Fe]	17/12	11.59^ab^ (5.11)	310.47^abc^ (104.92)	72.59^bc^ (32.25)	27.41^bc^ (32.25)	3.75^ab^ (6.90)	9.13^b^ (17.58)	14.52^b^ (23.76)	
DUN-75ppt-12L:12D-0[Fe]	10/1	10.60^ab^ (4.12)	273.80^bc^ (80.57)	97.54^bc^ (7.79)	2.46^bc^ (7.79)	0.00^b^	2.46^b^ (7.79)	0.00^b^	
DUN-75ppt-12L:12D-5[Fe]	10/3	11.00^ab^ (3.62)	229.20^abc^ (91.42)	77.82^bc^ (37.45)	21.18^bc^ (37.45)	1.56^ab^ (3.45)	3.92^b^ (12.39)	16.70^ab^ (26.93)	
DUN-75ppt-24L:00D-0[Fe]	32/22	13.25^a^ (5.36)	282.91^c^ (15.82)	64.69^bc^ (34.09)	35.31^bc^ (34.09)	5.82^b^ (16.62)	10.79^b^ (18.95)	18.70^ab^ (21.80)	
DUN-75ppt-24L:00D-5[Fe]	13/3	10.23^ab^ (4.28)	309.92^c^ (99.98)	85.65^bc^ (31.06)	14.35^bc^ (31.06)	2.10^b^ (7.58)	9.59^b^ (20.08)	2.66^b^ (9.59)	
TETRA-35ppt-12L:12D-0[Fe]	10/4	10.20^ab^ (3.36)	196.10^abc^ (131.49)	70.19^bc^ (39.47)	29.81^bc^ (39.47)	3.86^ab^ (12.22)	7.51^b^ (16.14)	18.44^ab^ (24.09)	
TETRA-35ppt-12L:12D-5[Fe]	14/3	10.29^ab^ (3.56)	276.64^abc^ (100.80)	83.09^bc^ (33.91)	16.91^bc^ (33.91)	0.00^b^	7.29^b^ (14.55)	9.62^b^ (19.40)	
TETRA-35ppt-24L:00D-0[Fe]	9/4	11.67^ab^ (4.03)	257.22^abc^ (145.38)	77.88^bc^ (27.29)	22.12^bc^ (27.29)	1.75^ab^ (4.93)	14.84^ab^ (19.72)	5.53^b^ (11.16)	
TETRA-35ppt-24L:00D-5[Fe]	15/5	11.60^ab^ (4.91)	280.20^abc^ (97.91)	91.87^bc^ (16.30)	8.13^bc^ (16.30)	2.08^b^ (7.26)	3.91^b^ (8.75)	2.15^b^ (8.24)	
TETRA-75ppt-12L:12D-0[Fe]	10/1	10.10^ab^ (3.78)	339.70^abc^ (131.83)	98.79^b^ (3.82)	1.21^b^ (3.82)	0.00^b^	0.00^b^	1.21^b^ (3.82)	
TETRA-75ppt-12L:12D-5[Fe]	10/2	10.70^ab^ (3.65)	276.20^abc^ (136.40)	93.57^bc^ (20.13)	6.43^bc^ (20.13)	0.00^b^	0.00^b^	6.43^b^ (20.13)	
TETRA-75ppt-24L:00D-0[Fe]	22/19	10.91^ab^ (3.94)	236.82^abc^ (117.11)	51.72^ac^ (38.85)	48.28^ac^ (38.85)	10.36^ab^ (12.80)	20.60^ab^ (20.71)	17.32^ab^ (24.83)	
TETRA-75ppt-24L:00D-5[Fe]	7/1	14.43^ab^ (10.69)	285.00^c^ (121.81)	99.92^bc^ (0.21)	0.08^bc^ (0.21)	0.00^b^	0.00^b^	0.08^b^ (0.21))	
Mean	235/129 (54.89%)	11.47 (4.61)	287.38 (132.26)	67.60 (38.05)	32.40 (38.05)	4.56 (10.48)	12.16 (18.80)	15.69 (22.54)	

Note. Different letters among treatments indicate significant differences (Dunn’s test, *p* < 0.05).

**TABLE 4:** Summary of reproductive parameters of 16 treatments recorded for the San Francisco Bay population.

Treatments	Total/oviparous females	Reproductive period at the 3rd brood (days)	Total offspring (number)	Offspring (%)				
				Nauplii	Encysted	Encysted 1st brood	Encysted 2nd brood	Encysted 3rd brood
		$\bar{x}\left(\pm \text{SD}\right)$	$\bar{x}\left(\pm \text{SD}\right)$	$\bar{x}\left(\pm \text{SD}\right)$	$\bar{x}\left(\pm \text{SD}\right)$	$\bar{x}\left(\pm \text{SD}\right)$	$\bar{x}\left(\pm \text{SD}\right)$	$\bar{x}\left(\pm \text{SD}\right)$
DUN-35ppt-12L:12D-0[Fe]	9/4	11.22^a^ (4.61)	384.00^a^ (173.25)	76.72^a^ (30.98)	23.28^a^ (30.98)	0.00^a^	9.72^a^ (19.45)	13.57^ab^ (20.55)
DUN-35ppt-12L:12D-5[Fe]	5/0	11.40^a^ (1.14)	199.20^a^ (79.09)	100.00^a^ (0.00)	0.00^a^ (0.00)	0.00^a^	0.00^a^	0.00^ab^
DUN-35ppt-24L:00D-0[Fe]	9/2	10.44^a^ (3.61)	286.33^a^ (191.95)	95.02^a^ (14.89)	4.98^a^ (14.89)	1.00^a^ (3.01)	3.96^a^ (11.88)	0.02^ab^ (0.06)
DUN-35ppt-24L:00D-5[Fe]	10/4	9.10^a^ (2.69)	477.20^a^ (268.46)	77.79^a^ (33.77)	22.21^a^ (33.77)	2.08^a^ (6.59)	6.27^a^ (13.44)	13.86^ab^ (21.67)
DUN-75ppt-12L:12D-0[Fe]	20/8	8.70^a^ (4.20)	343.80^a^ (173.96)	78.42^a^ (34.72)	21.58^a^ (34.72)	6.84^a^ (12.94)	5.73^a^ (14.62)	9.00^ab^ (17.42)
DUN-75ppt-12L:12D-5[Fe]	10/1	10.40^a^ (5.29)	318.30^a^ (147.20)	99.98^a^ (0.06)	0.02^a^ (0.06)	0.00^a^	0.00^a^	0.02^ab^ (0.06)
DUN-75ppt-24L:00D-0[Fe]	20/10	9.15^a^ (2.91)	357.25^a^ (120.51)	76.09^a^ (31.40)	23.91^a^ (31.40)	3.22^a^ (9.96)	8.90^a^ (20.05)	11.79^ab^ (20.98)
DUN-75ppt-24L:00D-5[Fe]	13/4	9.23^a^ (4.71)	308.08^a^ (150.70)	83.86^a^ (25.45)	16.14^a^ (25.45)	0.00^a^	1.82^a^ (6.56)	14.32^ab^ (22.59)
TETRA-35ppt-12L:12D-0[Fe]	4/2	8.50^a^ (5.07)	396.50^a^ (113.81)	79.35^a^ (24.70)	20.65^a^ (24.70)	0.00^a^	8.35^a^ (16.70)	12.30^ab^ (24.59)
TETRA-35ppt-12L:12D-5[Fe]	5/1	8.60^a^ (2.70)	323.20^a^ (124.37)	83.87^a^ (36.07)	16.13^a^ (36.07)	0.00^a^	7.65^a^ (17.11)	8.48^ab^ (18.96)
TETRA-35ppt-24L:00D-0[Fe]	9/1	12.78^a^ (6.22)	445.22^a^ (336.57)	94.04^a^ (17.89)	5.96^a^ (17.89)	2.20^a^ (6.59)	3.39^a^ (10.72)	0.00^b^
TETRA-35ppt-24L:00D-5[Fe]	6/4	9.67^a^ (2.34)	537.67^a^ (104.42)	67.77^a^ (39.73)	32.77^a^ (39.73)	0.00^a^	6.28^a^ (15.39)	31.87^a^ (25.87)
TETRA-75ppt-12L:12D-0[Fe]	13/1	7.46^a^ (2.37)	283.23^a^ (103.80)	92.31^a^ (27.74)	7.69^a^ (39.73)	2.29^a^ (8.24)	3.07^a^ (11.08)	2.33^ab^ (8.41)
TETRA-75ppt-12L:12D-5[Fe]	12/1	9.08^a^ (3.61)	445.58^a^ (245.69)	98.39^a^ (5.59)	1.61^a^ (5.59)	0.00^a^	1.61^a^ (5.59)	0.00^b^
TETRA-75ppt-24L:00D-0[Fe]	24/8	10.46^a^ (3.19)	308.08^a^ (189.42)	90.94^a^ (22.57)	9.06^a^ (22.57)	1.77^a^ (7.47)	5.79^a^ (12.53)	1.49^ab^ (6.42)
TETRA-75ppt-24L:00D-5[Fe]	19/2	8.79^a^ (1.61)	325.21^a^ (152.08)	98.45^a^ (6.49)	1.55^a^ (6.49)	1.55^a^ (6.49)	0.00^a^	0.00^b^
Mean	189/53 (28.04%)	9.58 (3.70)	371.83 (186.53)	87.26 (25.88)	12.74 (25.88)	1.87 (7.13)	4.46 (12.51)	6.41 (15.69)

Note. Different letters among treatments indicate significant differences (Dunn’s test, *p* < 0.05).

When comparing the data within each factor (Table 5), POE-3 in the BNE population presented significant differences for DIE, SAL, and IC (Mann–Whitney’s test, *p* < 0.05). In the case of the SFB population, only DIE significantly affected both POE and POE-3 (Mann–Whitney’s test, *p* < 0.05). In both populations, PHO did not have a significant effect (Mann–Whitney’s test, *p* > 0.05) (Table 5).

**TABLE 5:** Summary of reproductive parameters recorded for four factors in Barro Negro (BNE) and San Francisco Bay (SFB) populations.

Population	Factors		No. of females	Reproductive period at the 3rd brood (days)	Total offspring (number)	Offspring (%)				
						Nauplii	Encysted	Encysted 1st brood	Encysted 2nd brood	Encysted 3rd brood
				$\bar{x}\left(\pm \text{SD}\right)$	$\bar{x}\left(\pm \text{SD}\right)$	$\bar{x}\left(\pm \text{SD}\right)$	$\bar{x}\left(\pm \text{SD}\right)$	$\bar{x}\left(\pm \text{SD}\right)$	$\bar{x}\left(\pm \text{SD}\right)$	$\bar{x}\left(\pm \text{SD}\right)$
BNE	DIE	DUN	138	11.75^§^ (4.55)	**303.03** ^**§**^ (136.44)	**59.28** ^§^ (39.32)	**40.72** ^§^ (39.32)	**5.49** ^§^ (11.48)	**14.74** ^§^ (20.21)	**20.49** ^§^ (23.87)
		TETRA	97	11.07^§^ (4.69)	**265.11** ^**£**^ (128.96)	**79.43** ^£^ (32.77)	**20.57** ^£^ (32.77)	**3.23** ^£^ (8.72)	**8.48** ^£^ (15.87)	**8.86** ^£^ (18.61)
	SAL	35ppt	121	11.34^§^ (4.19)	298.00^§^ (142.77)	**59.40** ^§^ (39.77)	**40.60** ^§^ (39.77)	**5.08** ^§^ (9.61)	**15.45** ^§^ (19.75)	**20.07** ^§^ (23.78)
		75ppt	114	11.60^§^ (5.03)	276.11^§^ (124.66)	**76.30** ^£^ (34.11)	**23.70** ^£^ (34.11)	**4.01** ^£^ (11.33)	**8.66** ^£^ (17.07)	**11.03** ^£^ (20.24)
	IC	0[Fe]	136	**11.93** ^**§**^ (4.37)	288.13^§^ (145.63)	**56.82** ^§^ (39.29)	**43.18** ^§^ (39.29)	**6.66**§ (12.61)	**16.82**§ (20.62)	**19.70**§ (20.62)
		5[Fe]	99	**10.84** ^**£**^ (4.88)	286.35^§^ (118.03)	**82.40** ^£^ (30.66)	**17.60** ^£^ (30.66)	**1.67** ^£^ (5.30)	**5.75** ^£^ (13.52)	**10.18** ^£^ (20.84)
	PHO	12L:12D	109	10.96^§^ (3.56)	279.98^§^ (130.13)	63.93^§^ (41.56)	36.07^§^ (41.56)	3.79^§^ (8.87)	12.69^§^ (19.35)	19.58^§^ (24.18)
		24L:00D	126	10.90^§^ (5.33)	293.78^§^ (138.24)	70.76^§^ (34.50)	29.24^§^ (34.50)	5.22^§^ (11.68)	11.70^§^ (18.30)	12.32^§^ (20.54)
SFB	DIE	DUN	96	9.63^§^ (3.89)	362.70^§^ (174.13)	**83.37** ^§^ (28.42)	**16.63** ^§^ (28.42)	2.41^§^ (8.06)	5.23^§^ (14.16)	**8.99** ^§^ (17.99)
		TETRA	93	9.53^§^ (3.52)	381.26^§^ (199.32)	**91.27** ^£^ (22.33)	**8.73** ^£^ (22.33)	1.31^§^ (6.00)	3.67^§^ (10.62)	**3.75** ^£^ (12.07)
	SAL	35ppt	58	10.43^§^ (4.01)	403.05^§^ (223.84)	84.10^§^ (27.71)	15.90^§^ (27.71)	0.86^§^ (3.93)	5.67^§^ (13.64)	9.37^§^ (18.44)
		75ppt	131	9.20^§^ (3.51)	358.01^§^ (165.90)	88.66^§^ (25.05)	11.34^§^ (25.05)	2.31^§^ (8.11)	3.93^§^ (12.05)	5.10^§^ (14.10)
	IC	0[Fe]	109	9.73^§^ (3.96)	357.83^§^ (181.22)	85.16^§^ (27.82)	14.84^§^ (27.82)	**2.77** ^§^ (8.69)	**6.07** ^§^ (14.76)	5.99^§^ (14.96)
		5[Fe]	80	9.36^§^ (3.33)	390.91^§^ (193.59)	90.12^§^ (22.79)	9.88^§^ (22.79)	**0.63** ^£^ (3.91)	**2.27** ^£^ (8.29)	6.98^§^ (16.50)
	PHO	12L:12D	78	9.22^§^ (3.95)	345.04^§^ (170.56)	88.16^§^ (26.54)	11.84^§^ (26.54)	2.14^§^ (7.77)	4.27^§^ (12.34)	5.44^§^ (14.07)
		24L:00D	111	9.83^§^ (3.51)	390.66^§^ (197.63)	86.63^§^ (25.51)	13.37^§^ (25.51)	1.68^§^ (6.67)	4.60^§^ (12.74)	7.10^§^ (16.62)

Note. Different symbols between paired groups within each factor (§ and £) indicate a significant difference (boldface), according to the Mann–Whitney test (*p* < 0.05).

**FIGURE 2:**
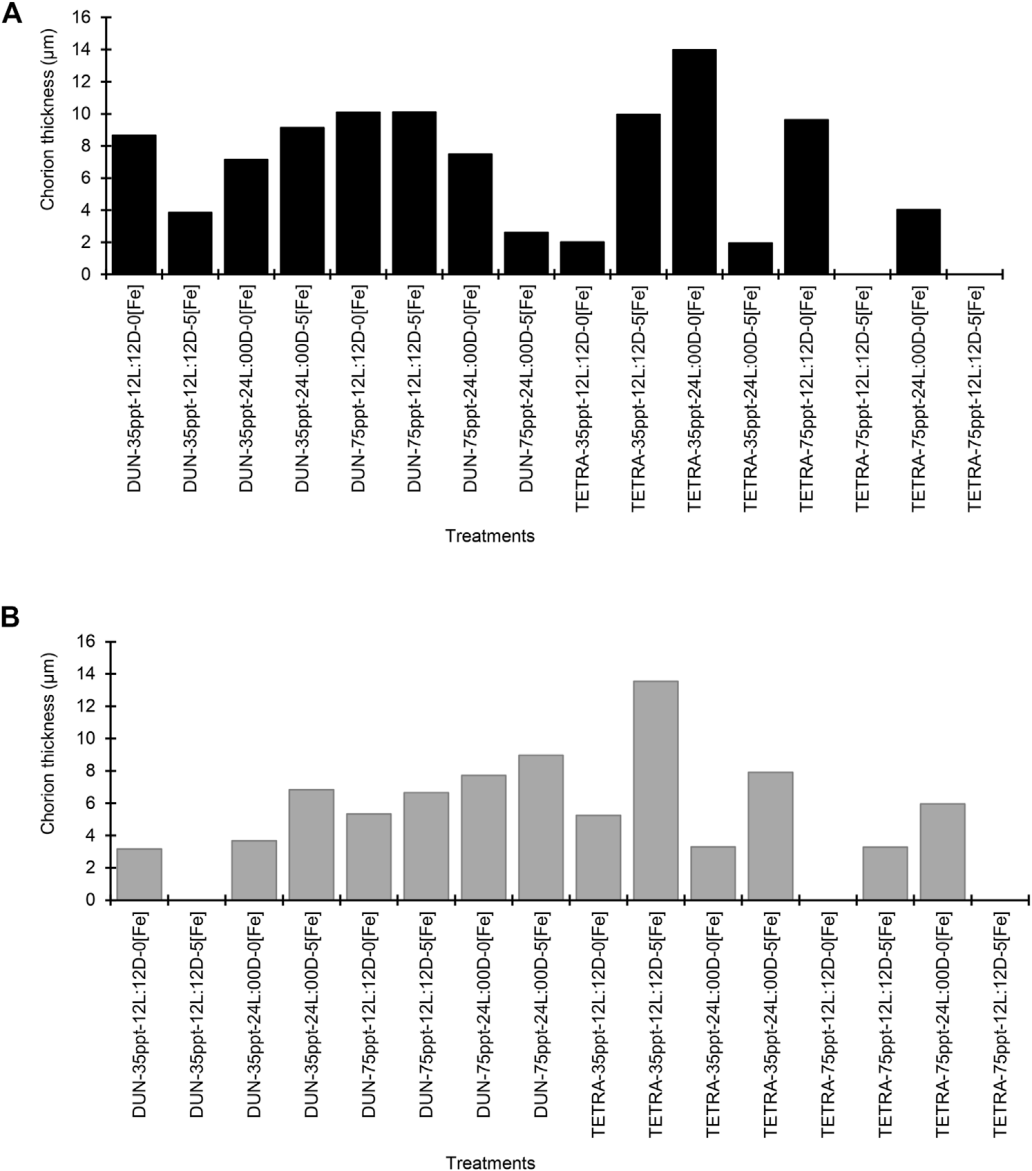
Chorion thickness (µm) in two populations of *Artemia franciscana* exposed to a combination of different diets, salinity, photoperiod, and iron concentration treatments. **(A)** Barro Negro population and **(B)** San Francisco Bay population.

For the BNE population, DHC varied between 212.40 ± 13.49 and 244.61 ± 15.33 μm (Supplementary Table S2), and this variation between treatments is significant in numerous comparisons (40/91 pairwise comparisons) (Dunn’s test, *p* < 0.05). In the case of DDC, this parameter varied between 198.97 ± 12.27 and 233.44 ± 10.75 μm, with significant variation between treatments in the majority of cases (50/91 pairwise comparisons) (Dunn’s test, *p* < 0.05). CT varied greatly, from 1.96 to 13.99 μm (Figure 2A). We observed considerable variation in the SFB population in both the DHC and DDC, which varied respectively between 202.98 ± 7.02 and 235.35 ± 12.18 μm and from 192.48 ± 9.60 to 217.83 ± 16.70 μm (Supplementary Table S2). In both parameters, differences between treatments were significant (DHC = 50/78 and DDC = 44/78 pairwise comparisons, Dunn’s test, *p* < 0.05). Similarly, CT varied in SFB from 3.17 to 13.54 μm (Figure 2B). When we compared data within each factor, the BNE population presented different CT values between the two culture conditions. For example, with the SAL treatment, CT presented a difference of 1.00 μm in thickness between the two treatments (greater in the 75 ppt condition). Similarly, in the PHO treatment, this parameter presented a difference of 1.20 μm in thickness (greater in PHO 12L:12D) (Supplementary Table S2). In the SFB population, the CT presented scarce variation between the two conditions of each factor, with the exception of IC (greater in the condition with iron).

### Gene Expression Analysis

**FIGURE 3:**
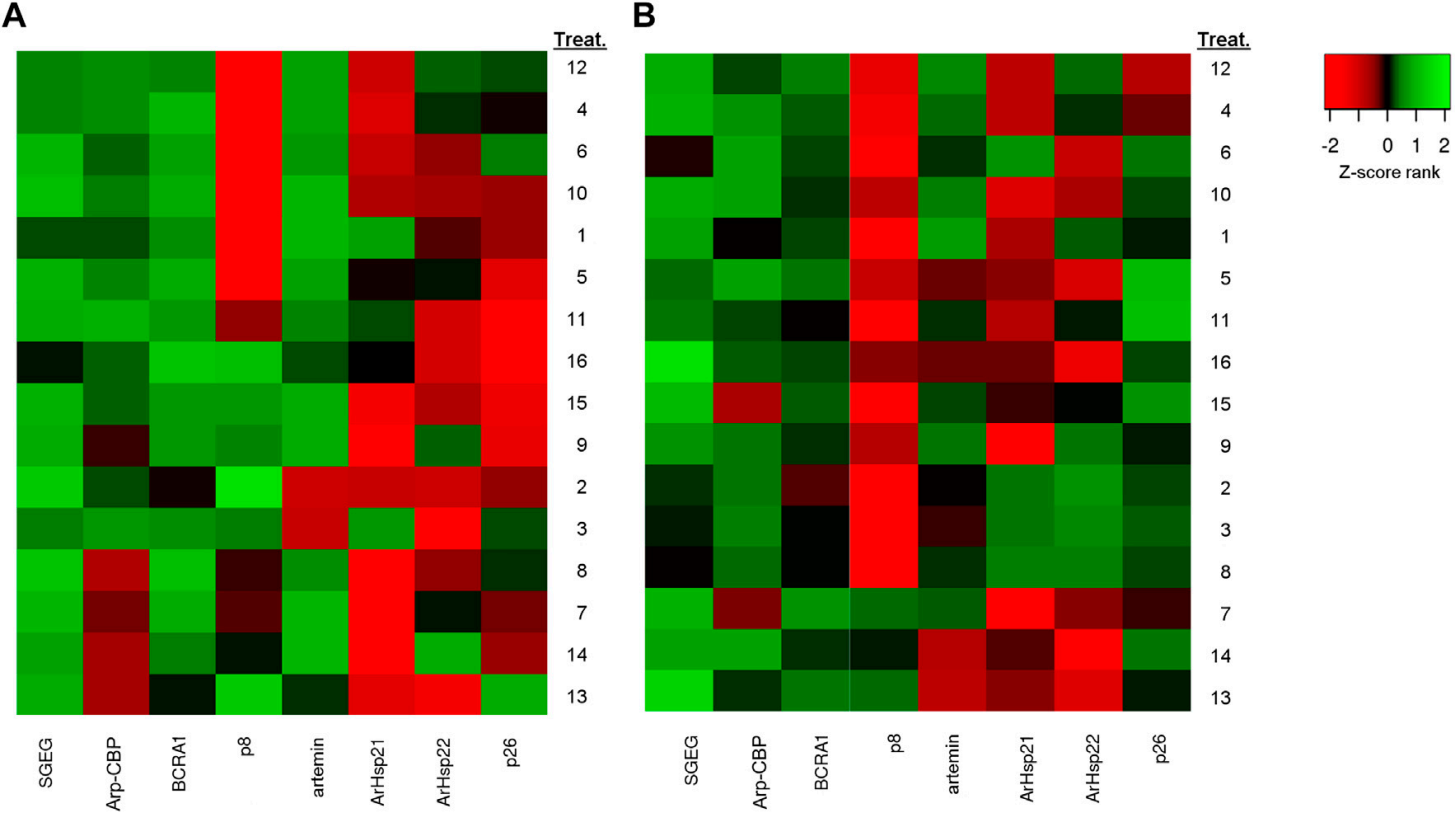
The differential gene expression analysis of *Artemia franciscana*. Heatmap of the differential genes between 16 treatments based on fold change values. **(A)** Barro Negro population (BNE) and **(B)** San Francisco Bay population (SFB). Red denotes downregulated expression, and green denotes upregulated expression. Treatment numbers are the same as those indicated in Table 2. To construct the heatmap, differences in the expression level of the genes were assessed after first normalizing expression level to those of *β-actin*, followed by log transformation (Log_2_).

The gene expression analysis in BNE revealed a significant differential expression between treatments for genes *SGEG*, *Arp-CBP*, *BRCA1*, *p*8, *artemin*, *ArHsp22*, and *p26* (Dunn’s test, *p* < 0.05), with pairwise differences between treatments of 6, 2, 10, 12, 1, 2, and 3 in a total of 120 comparisons, respectively (Table 6). We observed a similar pattern in the SFB population, although with fewer pairwise significant differences between treatments (*SGEG = 3*, *Arp-CBP = 1*, *BRCA1 = 2*, *p*8 = 2, *artemin = 2*, *ArHsp21* = 3, and *ArHsp22* = 7) (Dunn’s test, *p* < 0.05) (Table 7). Similarly, BNE presented a tendency towards gene up expression in most cases (38.28%) (Figure 3A); in contrast, SFB presented an up expression level of only 4.69%, given that in this population the tendency was mainly towards down expression (29.69%) (Figure 3B). The BNE population presented differentially expressed genes with marked changes in various treatments (>twofold) as compared to the SFB population. For example, in the DUN-75ppt-24L:00D-5[Fe], DUN-75ppt-12L:12D-5[Fe], DUN-35ppt-24L:00D-5[Fe], TETRA-35ppt-12L:12D-5[Fe], and TETRA-35ppt-24L:00D-5[Fe] treatments, six of the eight genes analyzed were up expressed. In the case of SFB, we did not observe this pattern of a strong change in expression, with the exception of the SFB-TETRA-75ppt-24L:00D-5[Fe] treatment, where only three genes showed an up expression level (*SGEG*, *Arp-CBP*, and *p26*).

**TABLE 6:** Gene expression level in the Barro Negro population.

Treatments	No. of females	Gene expression level							
		*SGEG*	*Arp-CBP*	*BRCA1*	*p8*	*artemin*	*ArHsp21*	*ArHsp22*	*p26*
		$\bar{x}\left(\pm \text{SD}\right)$	$\bar{x}\left(\pm \text{SD}\right)$	$\bar{x}\left(\pm \text{SD}\right)$	$\bar{x}\left(\pm \text{SD}\right)$	$\bar{x}\left(\pm \text{SD}\right)$	$\bar{x}\left(\pm \text{SD}\right)$	$\bar{x}\left(\pm \text{SD}\right)$	$\bar{x}\left(\pm \text{SD}\right)$
DUN-35ppt-12L:12D-0[Fe]	17	0.68^a^ (0.33)	0.75^a^ (0.45)	0.73^a^ (0.13)	0.28^a^ (0.22)↓	0.91^ab^ (0.29)	1.01^a^ (0.66)	0.92^ab^ (1.07)	0.64^abc^ (0.49)
DUN-35ppt-12L:12D-5[Fe]	4	1.03^ab^ (0.20)	0.62^ab^ (0.58)	0.26^a^ (0.07)↓	1.37^ab^ (0.09)	0.16^a^ (0.13)↓	0.14^a^ (0.09)↓	0.13^a^ (0.08)↓	0.18^ab^ (0.05)↓
DUN-35ppt-24L:00D-0[Fe]	7	0.98^ab^ (0.16)	1.18^ab^ (0.21)	1.14^ab^ (0.33)	0.97^ab^ (0.07)	0.50^a^ (0.33)	1.20^a^ (0.32)	0.36^ab^ (0.24)↓	0.92^abc^ (0.17)
DUN-35ppt-24L:00D-5[Fe]	10	3.47^ab^ (4.62)↑	2.96^b^ (3.25)↑	4.67^b^ (6.38)↑	0.70^ab^ (0.51)	3.23^c^ (3.16)↑	0.89^a^ (0.50)	4.20^ab^ (7.40)↑	2.69^abc^ (4.40)↑
DUN-75ppt-12L:12D-0[Fe]	5	3.04^b^ (1.80)↑	2.33^ab^ (2.08)↑	3.02^b^ (1.98)↑	1.71^ab^ (3.31)	2.71^abc^ (1.71)↑	1.85^a^ (2.52)	1.52^ab^ (0.65)	0.95^abc^ (0.84)
DUN-75ppt-12L:12D-5[Fe]	4	4.22^b^ (2.29)↑	3.24^ab^ (3.01)↑	4.01^b^ (3.00)↑	3.66^ab^ (4.19)↑	3.51^abc^ (2.32)↑	2.22^a^ (3.34)↑	1.61^ab^ (0.84)	2.95^abc^ (1.91)↑
DUN-75ppt-24L:00D-0[Fe]	4	2.47^ab^ (1.53)↑	0.83^ab^ (0.57)	2.56^ab^ (1.89)↑	0.73^ab^ (0.31)	2.85^abc^ (2.37)↑	0.19^a^ (0.14)↓	1.11^ab^ (0.57)	1.05^abc^ (0.83)
DUN-75ppt-24L:00D-5[Fe]	4	5.33^b^ (2.17)↑	3.87^ab^ (3.81)↑	5.31^b^ (2.36)↑	4.20^ab^ (3.38)↑	4.36^bc^ (1.79)↑	3.34^a^ (4.42)↑	3.06^b^ (2.00)↑	3.67^ac^ (1.45)↑
TETRA-35ppt-12L:12D-0[Fe]	5	3.65^ab^ (4.60)↑	1.34^ab^ (1.54)	3.04^ab^ (3.71)↑	2.66^ab^ (3.34)↑	3.98^abc^ (5.24)↑	0.25^a^ (0.32)↓	3.14^ab^ (5.07)↑	1.34^abc^ (2.31)
TETRA-35ppt-12L:12D-5[Fe]	10	3.41^b^ (1.86)↑	3.31^ab^ (3.08)↑	3.56^b^ (2.40)↑	3.25^ab^ (3.36)↑	3.55^c^ (2.00)↑	2.79^a^ (3.57)↑	1.83^ab^ (2.03)	1.78^abc^ (1.67)
TETRA-35ppt-24L:00D-0[Fe]	7	1.20^ab^ (0.22)	1.28^ab^ (0.27)	1.13^ab^ (0.19)	0.67^ab^ (0.33)	1.17^abc^ (0.56)	0.99^a^ (0.55)	0.51^ab^ (0.34)	0.33^b^ (0.23)↓
TETRA-35ppt-24L:00D-5[Fe]	7	2.55^b^ (1.22)↑	2.46^b^ (0.77)↑	2.43^b^ (1.16)↑	0.55^ab^ (0.54)	3.02^bc^ (1.54)↑	0.90^a^ (0.61)	2.57^b^ (1.57)↑	2.31^abc^ (1.40)↑
TETRA-75ppt-12L:12D-0[Fe]	6	2.34^b^ (0.71)↑	1.07^ab^ (0.55)	1.46^ab^ (0.40)	3.00^b^ (0.62)↑	1.52^abc^ (0.30)	0.76^a^ (0.44)	0.67^ab^ (0.49)	2.34^c^ (0.32)↑
TETRA-75ppt-12L:12D-5[Fe]	4	1.74^ab^ (0.32)	1.01^ab^ (0.62)	1.46^ab^ (0.27)	1.19^ab^ (0.16)	1.97^abc^ (0.30)	0.48^a^ (0.29)↓	1.91^ab^ (0.77)	0.93^abc^ (0.34)
TETRA-75ppt-24L:00D-0[Fe]	4	4.33^ab^ (5.01)↑	1.79^ab^ (1.31)	3.64^ab^ (4.52)↑	3.51^ab^ (4.09)↑	4.07^abc^ (4.99)↑	0.37^a^ (0.47)↓	0.70^ab^ (0.68)	0.73^abc^ (1.11)
TETRA-75ppt-24L:00D-5[Fe]	2	0.67^ab^ (0.46)	0.72^ab^ (0.18)	1.23^ab^ (0.52)	1.13^ab^ (0.33)	0.67^abc^ (0.18)	0.76^a^ (0.72)	0.40^ab^ (0.39)↓	0.36^abc^ (0.43)↓

Note. Different letters among treatments indicate significant differences (Dunn’s test, *p* < 0.05). Up arrow (↑) indicates genes with up expression, and down arrow (↓) indicates genes with down expression. Differences in the expression level of the genes were assessed after first normalizing expression level to those of *β-actin.*

**TABLE 7:** Gene expression level in the San Francisco Bay population.

Treatments	No. of females	Gene expression level							
		*SGEG*	*Arp-CBP*	*BRCA1*	*p8*	*artemin*	*ArHsp21*	*ArHsp22*	*p26*
		$\bar{x}\left(\pm \text{SD}\right)$	$\bar{x}\left(\pm \text{SD}\right)$	$\bar{x}\left(\pm \text{SD}\right)$	$\bar{x}\left(\pm \text{SD}\right)$	$\bar{x}\left(\pm \text{SD}\right)$	$\bar{x}\left(\pm \text{SD}\right)$	$\bar{x}\left(\pm \text{SD}\right)$	$\bar{x}\left(\pm \text{SD}\right)$
DUN-35ppt-12L:12D-0[Fe]	6	1.16^ab^ (0.15)	0.68^ab^ (0.48)	0.65^ab^ (0.03)	0.13^ab^ (0.06)↓	1.06^ab^ (0.21)	0.68^ab^ (0.69)	1.01^abc^ (0.83)	0.69^a^ (0.48)
DUN-35ppt-12L:12D-5[Fe]	5	0.95^ab^ (0.05)	1.34^ab^ (0.04)	0.58^ab^ (0.04)	0.09^ab^ (0.06)↓	0.73^ab^ (0.14)	1.44^a^ (0.14)	1.81^ab^ (0.12)	1.06^a^ (0.13)
DUN-35ppt-24L:00D-0[Fe]	2	0.77^ab^ (0.08)	1.32^ab^ (0.05)	0.68^ab^ (0.09)	0.06^ab^ (0.01)↓	0.55^ab^ (0.33)	1.20^ab^ (0.13)	1.46^abc^ (0.15)	1.01^a^ (0.06)
DUN-35ppt-24L:00D-5[Fe]	9	1.78^a^ (0.37)	1.40^ab^ (0.70)	0.91^ab^ (0.22)	0.17^ab^ (0.05)↓	1.00^ab^ (0.27)	0.51^ab^ (0.54)	0.82^abc^ (0.40)	0.78^a^ (0.67)
DUN-75ppt-12L:12D-0[Fe]	9	0.39^b^ (0.28)↓	0.71^ab^ (0.77)	0.45^a^ (0.30)↓	0.17^ab^ (0.11)↓	0.24^a^ (0.12)↓	0.24^ab^ (0.17)↓	0.15^c^ (0.08)↓	0.54^a^ (0.25)
DUN-75ppt-12L:12D-5[Fe]	4	0.93^ab^ (0.17)	2.48^a^ (0.43)↑	1.28^b^ (0.20)	0.24^ab^ (0.11)↓	1.20^ab^ (0.30)	2.06^a^ (0.50)↑	0.42^abc^ (0.15)↓	1.73^a^ (0.44)
DUN-75ppt-24L:00D-0[Fe]	4	1.37^ab^ (0.21)	0.37^ab^ (0.20)↓	1.06^ab^ (0.21)	0.89^a^ (0.47)	0.75^ab^ (0.27)	0.16^ab^ (0.16)↓	0.33^abc^ (0.10)↓	0.63^a^ (0.61)
DUN-75ppt-24L:00D-5[Fe]	4	0.91^ab^ (0.26)	1.68^ab^ (0.29)	1.00^ab^ (0.12)	0.08^b^ (0.06)↓	1.24^b^ (0.25)	2.00^a^ (0.39)↑	1.99^a^ (0.18)	1.30^a^ (0.11)
TETRA-35ppt-12L:12D-0[Fe]	4	1.61^a^ (0.21)	1.58^ab^ (1.01)	0.82^ab^ (0.24)	0.18^ab^ (0.02)↓	1.23^b^ (0.20)	0.06^b^ (0.02)↓	1.18^abc^ (0.24)	0.73^a^ (0.24)
TETRA-35ppt-12L:12D-5[Fe]	3	1.56^ab^ (0.18)	1.37^ab^ (0.12)	0.64^ab^ (0.02)	0.18^ab^ (0.01)↓	1.00^ab^ (0.29)	0.40^ab^ (0.61)↓	0.22^abc^ (0.04)↓	0.70^a^ (0.24)
TETRA-35ppt-24L:00D-0[Fe]	3	0.92^ab^ (0.75)	0.47^ab^ (0.27)↓	0.31^a^ (0.10)↓	0.07^ab^ (0.01)↓	0.50^ab^ (0.34)	0.16^ab^ (0.08)↓	0.40^abc^ (0.22)↓	1.06^a^ (0.43)
TETRA-35ppt-24L:00D-5[Fe]	6	1.40^ab^ (0.41)	1.19^ab^ (1.03)	1.01^ab^ (0.29)	0.18^ab^ (0.02)↓	1.07^ab^ (0.38)	0.50^ab^ (0.55)	1.07^abc^ (0.70)	0.60^a^ (0.47)
TETRA-75ppt-12L:12D-0[Fe]	4	1.38^ab^ (0.13)	0.45^ab^ (0.16)↓	0.57^ab^ (0.05)	0.97^ab^ (1.33)	0.16^ab^ (0.05)↓	0.22^ab^ (0.02)↓	0.14^c^ (0.12)↓	0.47^a^ (0.29)↓
TETRA-75ppt-12L:12D-5[Fe]	6	1.55^ab^ (1.71)	0.92^ab^ (0.62)	0.54^ab^ (0.33)	0.55^a^ (0.46)	0.28^ab^ (0.20)↓	0.41^ab^ (0.31)↓	0.17^c^ (0.12)↓	0.69^a^ (0.41)
TETRA-75ppt-24L:00D-0[Fe]	6	1.46^ab^ (0.86)	0.26^b^ (0.19)↓	0.61^ab^ (0.24)	0.12_ab_ (0.12)↓	0.77^ab^ (0.49)	0.42^ab^ (0.27)↓	0.55^abc^ (0.31)	1.02^a^ (0.67)
TETRA-75ppt-24L:00D-5[Fe]	5	3.55^a^ (2.37)↑	3.17^ab^ (5.43)↑	0.96^ab^ (0.49)	0.62^ab^ (0.58)	0.70^ab^ (0.63)	0.67^ab^ (0.56)	0.26b^c^ (0.26)↓	2.04^a^ (2.66)↑

Note. Different letters among treatments indicate significant differences (Dunn’s test, *p* < 0.05). Up arrow (↑) indicates genes with up expression, and down arrow (↓) indicates genes with down expression. Differences in the expression level of the genes were assessed after first normalizing expression level to those of *β-actin.*

In the case of down-expressed genes, in the BNE population, we only observed this expression pattern in some treatments, such as DUN-35ppt-12L:12D-5[Fe] (*BRCA1*, *ArHsp21*, *ArHsp22*, and *p26*) (Table 6), while in SFB, down-expressed genes were recorded in DUN-75ppt-12L:12D-0[Fe] (*SGEG*, *BRCA1*, *p8*, *artemin*, *ArHsp21*, and *ArHsp22*), DUN-75ppt-24L:00D-0[Fe] (*Arp-CBP*, *ArHsp21*, and *ArHsp22*), TETRA-35ppt-24L:00D-0[Fe] (Arp-CBP, *BRCA1*, *p8*, *ArHsp2*, and *ArHsp22*), TETRA-75ppt-12L:12D-0[Fe] (*Arp-CBP*, *artemin*, *ArHsp21*, *ArHsp22*, and *p*26), and TETRA-75ppt-12L:12D-5[Fe] (*artemin*, *ArHsp21*, and *ArHsp22*) (Table 7).

### Multiple Regression Analysis

In BNE, there were three plausible models with explanatory power (ΔAICc < 2) (Table 8). The top-ranked model for POE as the response variable included eight explanatory variables (*SGEG*, DIE, *artemin*, SAL, *Arp-CBP*, IC, *p8*, and *BRCA1*), being the most plausible model (ΔAICc = 0.00, *Wt* = 0.354) and explaining a high amount of the variation in the response variable (*R* ^2^ Adj = 0.57). The second top-ranked model included all aforementioned explanatory variables, except the *BRCA1* variable (*Wt* = 0.328), and the third top-ranked model did not include the variables *BRCA1* and p8 (*Wt* = 0.318).

**TABLE 8:** Multivariate model selection results in Barro Negro (BNE) and San Francisco Bay (SFB) populations.

Populations	Model	K	AIC	AICc	ΔAICc	*Wt*	Log likelihood
BNE	*SGEG*+DIE+*artemin*+SAL+*Arp-CBP*+IC+*p8*+*BRCA1*	8	938.62	941.09	0	0.354	−459.31
	*SGEG*+DIE+*artemin*+SAL+*Arp-CBP*+IC+*p8*	7	939.25	941.24	0.15	0.328	−460.62
	*SGEG*+DIE+*artemin*+SAL+*Arp-CBP*+IC	6	939.72	941.31	0.22	0.318	−461.86
	*SGEG*+DIE+*artemin*+SAL+*Arp-CBP*+IC+*p8*+*BRCA1*+*ArHsp22*	9	940.16	943.16	2.07		−459.08
SFB	SAL+A*rHsp21*+*artemin*	3	723.65	724.46	0	0.399	−356.83
	SAL+*ArHsp21*+*artemin*+*p8*	4	723.33	724.48	0.02	0.395	−355.67
	SAL+*ArHsp21*+*artemin*+*p8*+*ArHsp22*	5	724.23	725.78	1.32	0.206	−355.12
	SAL+*ArHsp21*	2	726.15	726.68	2.22		−359.07

Note. K, number of parameters for each mode; AIC, Akaike’s information criterion corrected for finite sample sizes; AICc, Akaike’s information criterion adjusted for small sample size; ΔAICc, relative difference in AICc, values compared with the top-ranked model; Wt, AIC, weight.

In the SFB, three models ranked the highest and with substantial explanatory power (ΔAICc < 2) but involved fewer predictor variables (3–5 variables) than in BNE (Table 8). The top-ranked model explained a low amount of variation in the response variable with the combination of SAL, *ArHsp21*, and *artemin* (*R* ^2^ Adj = 0.23), which corresponds to the best plausible model (ΔAICc = 0.00, *Wt* = 0.399). The second top-ranked model included SAL, *ArHsp21*, *artemin*, and *p8* (*Wt* = 0.395), and the third model included the same variables as the second top-ranked model in addition to the *ArHsp22* variable (*Wt* = 0.206).

## Discussion

### Incidence of Environmental Factors on Cyst Production

Our results support that diet, salinity, and iron concentration significantly affected the relative production of cysts in the populations analyzed, particularly in the BNE population (Table 5). This result is according to previous works that had identified these factors as important for the oviparous reproduction in *Artemia* (Versichele and Sorgeloos, 1980; Wang et al., 2017). For example, in the case of salinity, the literature indicates that low salinities (<90 ppt) are important for increasing cyst production (Versichele and Sorgeloos, 1980; Berthélémy-Okazaki and Hedgecock, 1987), although other studies show contradictory results, since high salinity (>100 ppt) shows positive effects (Castro-Mejía et al., 2013; Wang et al., 2017). Our results support the former finding, given that the lowest salinity used in our study (35 ppt), in comparison with the highest salinity (75 ppt), consistently surpassed twice the relative cyst production. We expected that iron addition to the culture (5 mg/L) would produce a positive effect on cyst production in the two analyzed populations, as the literature indicated (Dutrieu 1960; Versichele and Sorgeloos, 1980), since this factor is positively correlated with cyst production per female in *Artemia*. However, a contrary effect was observed in the populations studied, given that cyst production decreased significantly (approximately −50%) with the aforementioned iron concentration. We believe that these differences may be due to the type of iron used, which in our case was ferrous sulfate and not Fe-EDTA, as was indicated in previous studies (Versichele and Sorgeloos, 1980). However, given that iron availability in the culture media should be similar when both ferrous compounds are used, it is likely that a population-specific factor may be involved in the reduced cyst production. Further comparative assays will require to test if this factor is involved. Since different diets substantially modify cyst production in *Artemia* (Versichele and Sorgeloos, 1980; Vartak and Joshi, 2002; Esteve and ElMasri, 2007), we explored the use of two microalgae diets with different nutritional compositions (*D. tertiolecta* and *T. suecica*), to obtain evidence on the effect of the diet quality in our assay. The analysis of microalgae diet merit to be studied due to this is a key factor to improve cyst production in *Artemia*, whose implementation in intensive culture is relatively simple. We found evidence that cyst production was significantly different when these microalgae diets were used. In fact, the *D. tertiolecta* diet almost duplicated the relative quantity of cysts in comparison to the alternative *T. suecica* diet. Thus, this result could be related to the nutritional composition of these microalgae, given that these microalgae have a different proportion of fatty acid, carotene, protein, and carbohydrate. For example, *T. suecica* has higher content of β-carotenoids than *D. tertiolecta* (50.6 vs. 39.5 pg/cell) (Cifuentes et al., 1996; Moreno Bayon, 2010; López-Elías et al., 2013). In addition, *T. suecica* has around twice of protein (52.1 vs. 20.0 pg/cell) and carbohydrate (20.2 vs. 12.2 pg/cell) content than *D. tertiolecta* (Lavens and Sorgeloos, 1996). Some studies had shown that certain photoperiod conditions can induce greater cyst production in *Artemia* (Sorgeloos et al., 1976; Berthélémy-Okazaki and Hedgecock, 1987; Nambu et al., 2004; Wang et al., 2017). For example, the 8L:16D condition was reported by Sorgeloos et al. (1976). In addition, recent studies support this previous finding, since the 10L:14D condition tends to produce increased cyst production (Nambu et al., 2004), as occurs with the light decreasing from 18 to 6 h (Wang et al., 2017). Of note, however, is that in both studies, this effect would depend, to a certain extent, on temperature variation (19°C–28°C). We did not observe this effect in our study, since when the photoperiod decreased from 24 to 12 h, the percent of offspring encysted at third brood did not differ significantly, either in BNE or in SFB (Table 5). However, this result could be taken with caution, since in our assays no short light regimen was assessed.

### Environmental Factors That Affect Cyst Diameter

We observed that in SFB, salinity and iron concentration affected the chorion thickness the most, as compared to BNE. This result is not rare given that available data in *Artemia* indicate that there is wide interpopulation variation in the chorion thickness (Sorgeloos et al., 1986). There is little information on factors that affect the chorion thickness in *Artemia* (Wang et al., 2019). In this work, we provide evidence on the significant effect of salinity and iron concentration on the chorion thickness in two *A. franciscana* populations. Our results coincide with Wang et al. (2019), given that they found that increased salinity had a negative effect on the chorion thickness. Moreover, since these authors reported that variation in iron concentration also had an effect on the chorion thickness, which was thinner at 5 mg of Fe^2+^/L and thicker at higher iron concentrations, our result also concurs with these findings. Several factors have been identified on the formation of the chorion, such as secretion of hematin from the shell gland, low oxygen concentration in the environment (Dutrieu 1960), and an increase in salinity (Browne et al., 1988; Browne and Wanigasekera, 2000; Abatzopoulos et al., 2003; Medina et al., 2007). Moreover, *SGEG* and *Arp-CBP* genes also participated in chorion formation in *Artemia* (Dai et al., 2011; Qiu et al., 2007), because these genes are expressed under high salinity or ion concentration. In our case, results support that both genes could be related to chorion thickening, since these genes presented an up expression pattern when increasing salinity and ion concentration were used (Table 6).

### Gene Expression Analysis

In *Artemia*, various genes associated with response to environmental changes have been identified, such as *BRCA1*, *p8*, *artemin*, *ArHsp21*, *ArHsp22*, and *p26* (Qiu et al., 2007; Qiu and MacRae, 2008a, Qiu and MacRae, 2008b; Liu et al., 2009; Dai et al., 2011; King et al., 2013; King et al., 2014; Zhou et al., 2014; Ye et al., 2017). Specifically, these genes have been related to the diapause reproductive mode, such as *BRCA1* and *p8* (Qiu et al., 2007; Qiu and MacRae, 2007), and also with cyst resistance to stress, such as *artemin* (King et al., 2014), *SGEG* (Liu et al., 2009), *Arp-CBP* (Wen-Ming et al., 2013), and *SGEG*1 and *SGEG*2 (Dai et al., 2011). In the latter two cases, their function would be related to the formation of the chorion outer layer, which protects the encysted embryo from adverse environments (Dai et al., 2011). These gene expression studies represent significant progress made with regard to the identification of some genes in *Artemia* associated with adaptation to inhospitable environments. However, their usefulness has still not been tested in an applied field, particularly with regard to the prediction of reproductive mode leading to diapause under controlled culture conditions. From a commercial point of view, controlled *Artemia* cyst production is important, given that the cysts have a high production value in fish larviculture, for example, in native marine or freshwater fish with a high commercial value, which is being developed in Central America (Abdo-de la Parra et al., 2015; Palma-Cancino et al., 2019) and South America (Fabregat et al., 2015; Luz and Portella, 2015; Dos Santos et al., 2016). In our study, we contribute new information with regard to this topic, by evaluating the expression level of eight previously described genes, in order to analyze their relationship with the diapause reproductive mode in a Chilean population characterized by high cyst production.

Our results of multiple regression analyses indicate that in BNE, five genes (*SGEG*, *artemin*, *Arp-CBP*, *p8*, and *BRCA1*) and three environmental factors (DIE, SAL, and IC) are important predictor variables for the POE response variable, given that all of them were included in the highest-ranking models. On the contrary, in SFB, only two genes (Ar*Hsp21* and *artemin*) and one environmental factor (SAL) were important explanatory variables in the highest-ranking models. In BNE, this result suggests that these variables are possibly causally linked to POE and that the identified genes could be candidates for monitoring the reproductive mode leading to the diapause of this population. In fact, these genes tend to be up expressed in BNE; for example, *SGEG* and *artemin* were differentially expressed in 10 and 9 out of 16 treatments, respectively.

Literature indicates that *SGEG* and *Arp-CBP* genes are important for chorion formation (Liu et al., 2009; Dai et al., 2011). In fact, Liu et al. (2009) concluded that *SGEG* gene expression is important for chorion formation since inhibition of this gene by *RNAi* produced cysts with a low resistance to environmental stressors, such as constant aeration, high salinity (6M), and osmotic shock. In our case, we observed in BNE that salinity, iron concentration, and photoperiod affect the chorion thickness (Supplementary Table S2). This result is in accordance with multiple regression analysis given that salinity and iron concentration variables in combination with *SGEG* and *Arp-CBP* genes affected the POE response variable. In fact, both genes showed an up expression pattern in several treatments in the BNE population (Figure 3A). In addition, the up expression pattern of *p8* and *BRCA1* genes analyzed in BNE (Figure 3A) concur with information provided in previous studies, given that Qiu et al. (2007), through transcriptomic studies, reported that these genes are up expressed in oviparous ovisacs. Similarly, other studies performed using semiquantitative RT-PCR reported that *p8* gene is also up expressed in embryos destined for diapause (Qiu et al., 2007). In contrast, in SFB, these genes were mostly down expressed, which may be related to the low cyst production observed for this population. On the other hand, the inclusion of *ArHsp21* gene as an explanatory variable in all top-ranked models of the regression analysis for SFB is an interesting result given that this gene encodes for a chaperone or heat shock protein that plays a minor role in diapause formation (Qiu and MacRae, 2008a). In fact, the literature indicates that *ArHsp21* gene has a diminished expression in embryos not destined to diapause since it has transient expression in larvae (Qiu and MacRae, 2008a). Other studies indicate that the failure expression of *ArHsp21* gene, along with related genes that encode for other small heat shock proteins, affects the survival of cysts in *Artemia* (Tan and MacRae, 2018). Future studies will be required to test whether there is diminished viability of cysts produced by the SFB population.

## Conclusion

In conclusion, results obtained with the treatments used indicate that diet, salinity, and iron concentration are important factors for cyst production in the two populations analyzed, mainly in the BNE population. Furthermore, in the BNE population, salinity and photoperiod significantly change the chorion thickness, while in the SFB population, all factors analyzed affected this parameter. Likewise, in BNE, we observed significant differential gene expression in the majority of genes analyzed (*SGEG*, *Arp-CBP*, *artemin*, *BRCA1*, *p8*, *ArHsp22*, and *p26*) with up expression in most cases, while in SFB, various genes also presented changes in their expression level (*SGEG*, *Arp-CBP*, *artemin*, *BRCA1*, *p8*, *ArHsp21*, and *ArHsp22*), but with a marked tendency towards down expression. The up gene expression trend observed in BNE, in comparison with SFB, could indicate that this gene expression pattern is associated with populations characterized by markedly oviparous reproduction. The multiple regression analysis supports that five of these genes (*SGEG*, *artemin*, *Arp-CBP*, *p8*, and *BRCA1*) could be important predictor variables for cyst production in this population, given that all of them were included in the highest-ranking model. Thus, this gene expression pattern could be useful for monitoring the reproductive mode leading to diapause in *Artemia*, either to assess cyst production in controlled cultures or to diagnose the existence of natural populations or strains with a high cyst production capacity. However, the scaling up of the results of this work to the productive level requires the confirmation of the pattern of gene expression observed through more reliable techniques, such as quantitative real-time PCR. In addition, the response for cyst production with a wider range of salinities and photoperiod conditions should be evaluated in order to find a more optimal culture condition. In this way, future studies that resolve these restrictions will make it possible to apply these results to identify highly cyst-producing strains of *Artemia*. Thus, the use of strains of this class in controlled systems may represent an interesting alternative for the production of cysts for commercial purposes.

## Data Availability

The original contributions presented in the study are included in the article/supplementary material, Further inquiries can be directed to the corresponding author.

## References

[B1] Abatzopoulos T. J. El-Bermawi N. Vasdekis C. Baxevanis A. D. Sorgeloos P. (2003). Effects of Salinity and Temperature on Reproductive and Life Span Characteristics of Clonal *Artemia*. (International Study on *Artemia*. LXVI). Hydrobiologia 492, 191–199. 10.1023/A:1024826702830

[B2] Abdo-de la Parra M. I. Rodríguez-Ibarra L. E. Rodríguez-Montes de Oca G. Velasco-Blanco G. Ibarra-Casto L. (2015). State of Art for Larval Rearing of Spotted Rose Snapper (Lutjanus Guttatus). Lat. Am. J. Aquat. Res. 43 (3), 415–423. 10.3856/vol43-issue3-fulltext-3

[B3] Babicki S. Arndt D. Marcu A. Liang Y. Grant J. R. Maciejewski A. (2016). Heatmapper: Web-Enabled Heat Mapping for All. Nucleic Acids Res. 44, W147–W153. 10.1093/nar/gkw419 27190236 PMC4987948

[B4] Bartoń K. (2013). MuMIn: Multi‐model Inference. R Package Version 1.9.13. Vienna, Austria: Compr. R Arch. Netw. (CRAN).

[B5] Berthélémy-Okazaki N. J. Hedgecock D. (1987). “Effect of Environmental Factors on Cyst Formation in the Brine Shrimp *Artemia* ,” in Artemia Research and its Applications. Editors Sorgeloos P. Bengtson D. A. Decleir W. Jaspers E. (Wetteren Belgium: Universa Press), 3, 167–182.

[B6] Browne R. A. Davis L. E. Sallee S. E. (1988). Effects of Temperature and Relative Fitness of Sexual and Asexual Brine Shrimp *Artemia* . J. Exp. Mar. Biol. Ecol. 124, 1–20. 10.1016/0022-0981(88)90201-8

[B7] Browne R. A. (1980). Reproductive Pattern and Mode in the Brine Shrimp. Ecology 61 (3), 466–470. 10.2307/1937408

[B8] Browne R. A. Sallee S. E. Grosch D. S. Segreti W. O. Purser S. M. (1984). Partitioning Genetic and Environmental Components of Reproduction and Lifespan in *Artemia* . Ecology 65 (3), 949–960. 10.2307/1938067

[B9] Browne R. A. Wanigasekera G. (2000). Combined Effects of Salinity and Temperature on Survival and Reproduction of Five Species of *Artemia* . J. Exp. Mar. Biol. Ecol. 244, 29–44. 10.1016/S0022-0981(99)00125-2

[B10] Burnham K. Anderson D. (2002). Model Selection and Multimodel Inference. A Practical Information-Theoretic Approach. New York: Springer-Verlag.

[B11] Capuano E. Boerrigter-Eenling R. van der Veer G. van Ruth S. M. (2013). Analytical Authentication of Organic Products: an Overview of Markers. J. Sci. Food Agric. 93, 12–28. 10.1002/jsfa.5914 23070660

[B12] Castro-Mejía J. Castro-Mejía G. Ramírez-Orozco D. I. de Lara Andrade R. Monroy-Dosta M. C. Ramírez-Torrez J. A. (2013). Sobrevivencia y características reproductivas de las poblaciones de *Artemia franciscana* Kellogg. 1906 provenientes de Yucatán, México, cultivadas a diferentes salinidades (40. 60. 80. 100 y 120 gL^-1^). Revista Digital Del. Departamento El Hombre y su Ambiente 2 (4), 61–72.

[B13] Chen T. Villeneuve T. S. Garant K. A. Amons R. MacRae T. H. (2007). Functional Characterization of Artemin, a Ferritin Homolog Synthesized in *Artemia* embryos during Encystment and Diapause. FEBS J. 274, 1093–1101. 10.1111/j.1742-4658.2007.05659.x 17257268

[B14] Chen Y.-r. Jiang T. Zhu J. Xie Y.-c. Tan Z.-c. Chen Y.-h. (2017). Transcriptome Sequencing Reveals Potential Mechanisms of Diapause Preparation in Bivoltine Silkworm *Bombyx mori* (Lepidoptera: Bombycidae). Comp. Biochem. Physiol. D: Genomics Proteomics 24, 68–78. 10.1016/j.cbd.2017.07.003 28850878

[B15] Chomczynski P. (1993). A Reagent for the Single-step Simultaneous Isolation of RNA, DNA and Proteins from Cell and Tissue Samples. Biotechniques 15 (3), 532–537. 7692896

[B16] Cifuentes A. S. González M. Parra O. Zúñiga M. (1996). Cultivo de cepas de *Dunaliella salina* (Teodoresco 1905) en diferentes medios bajo condiciones de laboratorio. Revista Chilena de Historia Nat. 69, 105–112.

[B17] Clegg J. S. Conte F. (1980). “A Review of the Cellular and Developmental Biology of *Artemia* ,” in The Brine Shrimp. Artemia. Editors Persoone G. P. Sorgeloos P. Roels O. Jaspers E. (Wetteren, Belgium: Universa Press), 2, 11–54.

[B18] Clegg J. S. Gajardo G. (2009). Two Highly Diverged New World *Artemia* Species, *A. franciscana* and *A. persimilis*, from Contrasting Hypersaline Habitats Express a Conserved Stress Protein Complement. Comp. Biochem. Physiol. A: Mol. Integr. Physiol. 153 (4), 451–456. 10.1016/j.cbpa.2009.04.613 19379819

[B19] Clegg J. S. Jackson S. A. Warner A. H. (1994). Extensive Intracellular Translocations of a Major Protein Accompany Anoxia in Embryos of *Artemia franciscana* . Exp. Cel Res. 212, 77–83. 10.1006/excr.1994.1120 8174644

[B20] Dai L. Chen D.-F. Liu Y.-L. Zhao Y. Yang F. Yang J.-S. (2011). Extracellular Matrix Peptides of *Artemia* Cyst Shell Participate in Protecting Encysted Embryos from Extreme Environments. PLoS ONE 6 (6), e20187. 10.1371/journal.pone.0020187 21673998 PMC3108945

[B21] dos Santos J. C. E. Pedreira M. M. Luz R. K. (2016). Feeding Frequency in Pacamã Larviculture. Rev. Caatinga 29 (2), 512–518. 10.1590/1983-21252016v29n230rc

[B22] Dutrieu J. (1960). Observations biochimiques et physiologique sur le devéloppement d’*Artemia* salina Leach. Arch. Zool. Exp. Gén. 99, 1–134.

[B23] Esteve M. ElMasri R. (2007). Sobrevivencia, producción y calidad de quistes de *Artemia* (cepa Araya), Edo. Sucre, Venezuela, bajo combinaciones de tres dietas y tres salinidades. Saber. Universidad de Oriente. Venezuela. 19 (1), 3–13. (ISSN: 1315-0162).

[B24] Fabregat T. E. H. P. Damian J. Fialho N. S. Costa D. Broggi J. A. Pereira R. G. (2015). Toxicidade aguda ao sal comum e larvicultura intensiva Do jundiá *Rhamdia quelen* em água salobra. Arq. Bras. Med. Vet. Zootec. 67 (2), 547–554. 10.1590/1678-7660

[B25] Gajardo G. Colihueque N. Parraguez M. Sorgeloos P. (1998). International Study on *Artemia* LVIII. Morphologic Differentiation and Reproductive Isolation of *Artemia* Populations from South America. Int. J. Salt Lake Res. 7, 133–151. 10.1007/bf02441883

[B26] Gajardo G. Parraguéz M. Beardmore J. A. Sorgeloos P. (2001). Reproduction in the Brine Shrimp *Artemia* : Evolutionary Relevance of Laboratory Cross‐fertility Tests. J. Zoolog. 253, 25–32. 10.1017/S0952836901000036

[B27] Jackson S. A. Clegg J. S. (1996). Ontogeny of Low Molecular Weight Stress Protein p26 during Early Development of the Brine Shrimp, *Artemia franciscana* . Dev. Growth Differ. 38, 153–160. 10.1046/j.1440-169X.1996.t01-1-00004.x 37280891

[B28] King A. M. MacRae T. H. (2012). The Small Heat Shock Protein p26 Aids Development of Encysting *Artemia* Embryos, Prevents Spontaneous Diapause Termination and Protects against Stress. PLoS One 7 (8), e43723. 10.1371/journal.pone.0043723 22952748 PMC3428274

[B29] King A. M. Toxopeus J. MacRae T. H. (2014). Artemin, a Diapause-specific Chaperone, Contributes to the Stress Tolerance of *Artemia* Cysts and Influences Their Release from Females. J. Exp. Biol. 217, 1719–1724. 10.1242/jeb.100081 24526727

[B30] King A. M. Toxopeus J. MacRae T. H. (2013). Functional Differentiation of Small Heat Shock Proteins in Diapause-destined *Artemia* Embryos. FEBS J. 280, 4761–4772. 10.1111/febs.12442 23879561

[B31] Lavens P. Sorgeloos P. (1996). Manual on the Production and Use of Live Food for Aquaculture. Belgium: State University of Ghent.

[B32] Liang P. MacRae T. H. (1999). The Synthesis of a Small Heat Shock/α-Crystallin Protein in *Artemia* and its Relationship to Stress Tolerance during Development. Develop. Biol. 207 (2), 445–456. 10.1006/dbio.1998.9138 10068475

[B33] Liu Y.-L. Zhao Y. Dai Z.-M. Chen H.-M. Yang W.-J. (2009). Formation of Diapause Cyst Shell in Brine Shrimp, *Artemia parthenogenetica*, and its Resistance Role in Environmental Stresses. J. Biol. Chem. 284 (25), 16931–16938. 10.1074/jbc.M109.004051 19395704 PMC2719330

[B34] López-Elías J. A. Fimbres-Olivarría D. Medina-Juárez L. A. Miranda-Baeza A. Martínez-Córdova L. R. Molina-Quijada D. M. A. (2013). Producción de biomasa y carotenoides de *Dunaliella tertiolecta* en medios limitados en nitrógeno. FYTON 82, 23–30. ISSN 0031 9457.

[B35] Luz R. K. Portella M. C. (2015). Effect of Prey Concentrations and Feed Training on Production of *Hoplias Lacerdae* Juvenile. Acad. Bras. Ciênc. 87 (2), 1125–1132. 10.1590/0001-3765201520140412 25860973

[B36] MacRae T. H. (2003). Molecular Chaperones, Stress Resistance and Development in *Artemia franciscana* . Semin. Cel Develop. Biol. 14, 251–258. 10.1016/j.semcdb.2003.09.019 14986854

[B37] MacRae T. H. (2016). Stress Tolerance during Diapause and Quiescence of the Brine Shrimp, *Artemia* . Cell Stress and Chaperones 21, 9–18. 10.1007/s12192-015-0635-7 26334984 PMC4679736

[B38] Medina G. R. Goenaga J. Hontoria F. Cohen G. Amat F. (2007). Effects of Temperature and Salinity on Prereproductive Life Span and Reproductive Traits of Two Species of *Artemia* (Branchiopoda, Anostraca) from Argentina: *Artemia franciscana* and *A. persimilis* . Hydrobiologia 579, 41–53. 10.1007/s10750-006-0361-3

[B39] Moraga C. P. Ávila P. R. Vilaxa O. A. (2015). Salinidad y temperatura óptimas para reproducción ovípara y desarrollo de *Artemia franciscana* . Idesia 33 (1), 85–92. 10.4067/S0718-34292015000100009

[B40] Moreno Bayon J. P. (2010). “Evaluación del crecimiento y carotenogénesis de cuatro cepas de microalgas marinas bajo condiciones de estrés por iluminación a temperaturas y salinidades constantes,” in Tesis de Posgrado, Ciencias Biológicas y de la Salud (México: Universidad de Sonora). http://hdl.handle.net/20.500.12984/754.

[B41] Nambu Z. Tanaka S. Nambu F. (2004). Influence of Photoperiod and Temperature on Reproductive Mode in the Brine Shrimp, *Artemia franciscana* . J. Exp. Zool. 301A, 542–546. 10.1002/jez.a.80 15181648

[B42] Ojima N. Yamashita M. Watabe S. (2005). Quantitative mRNA Expression Profiling of Heat-Shock Protein Families in Rainbow trout Cells. Biochem. Biophysical Res. Commun. 329 (1), 51–57. 10.1016/j.bbrc.2005.01.097 15721272

[B43] Overturf K. (2009). “Quantitative PCR,” in Molecular Research in Aquaculture. Editor Overturf K. (Oxford, UK: Wiley-Blackwell), 39–61. 10.1002/9780813807379.ch3

[B44] Palma-Cancino D. J. Martínez-García R. Álvarez-González C. A. Camarillo-Coop S. Peña-Marin E. S. (2019). Esquemas de alimentación para larvicultura de pejelagarto (Atractosteus tropicus Gill): crecimiento, supervivencia y canibalismo. Ecosist. Recur. Agropec. 6 (17), 273–281. 10.19136/era.a6n17.2092

[B45] Peykaran Mana N. Vahabzadeh H. Hafezieh M. Seidgar M. Shoa Hasani A. Yazdani Sadati M. A. (2011). Biometrical Characters of *Artemia* from Four Iranian Regions. IJFS 10 (2), 294–303.

[B46] Pruisscher P. Nylin S. Gotthard K. Wheat C. W. (2018). Genetic Variation Underlying Local Adaptation of Diapause Induction along a Cline in a Butterfly. Mol. Ecol. 27, 3613–3626. 10.1111/mec.14829 30105798

[B47] Qiu Z. MacRae T. H. (2008a). *ArHsp*21, a Developmentally Regulated Small Heat-Shock Protein Synthesized in Diapausing Embryos of *Artemia franciscana* . Biochem. J. 411 (3), 605–611. 10.1042/BJ20071472 18095938

[B48] Qiu Z. MacRae T. H. (2008b). *ArHsp*22, a Developmentally Regulated Small Heat Shock Protein Produced in Diapause-Destined *Artemia* Embryos, Is Stress Inducible in Adults. FEBS J. 275 (14), 3556–3566. 10.1111/j.1742-4658.2008.06501.x 18537825

[B49] Qiu Z. MacRae T. H. (2007). Developmentally Regulated Synthesis of *P*8, a Stress-Associated Transcription Cofactor, in Diapause-Destined Embryos of *Artemia franciscana* . Cell Stress Chaper 12 (3), 255–264. 10.1379/csc-275.1 PMC1971234 17915558

[B50] Qiu Z. Tsoi S. C. M. MacRae T. H. (2007). Gene Expression in Diapause-Destined Embryos of the Crustacean, *Artemia franciscana* . Mech. Develop. 124, 856–867. 10.1016/j.mod.2007.09.001 17950581

[B51] Ren S. Hao Y.-J. Chen B. Yin Y.-P. (2018). Global Transcriptome Sequencing Reveals Molecular Profiles of Summer Diapause Induction Stage of Onion Maggot, *Delia antiqua* (Diptera: Anthomyiidae). Genomes. Genet. 8 (1), 207–217. 10.1534/g3.117.300393 PMC5765349 29158334

[B52] Rice W. R. (1989). Analyzing Tables of Statistical Tests. Evolution 43 (1), 223–225. 10.2307/2409177 28568501

[B53] Shirdhankar M. M. Thomas P. C. Barve S. K. (2004). Phenotypic Estimates and Heritability Values of *Artemia franciscana* . Aquac. Res. 35, 35–39. 10.1111/j.1365-2109.2004.00976.x

[B54] Smyth G. K. Yang Y. H. Speed T. (2003). Statistical Issues in cDNA Microarray Data Analysis. Methods Mol. Biol. 224, 111–136. 10.1385/1-59259-364-X:111 12710670

[B55] Sokal R. R. Rohlf F. J. (1995). Biometry: The Principles and Practice of Statistics in Biological Research. New York: Freeman.

[B56] Sorgeloos P. Baeza-Mesa M. Benijts F. Persoone G. (1976). “Research on the Culturing of the Brine Shrimp *Artemia salina* L. At the State University of Ghent (Belgium),” in Proceedings of the 10th European Symposium on Marine Biology. Editors Persoone G. Jaspers E. (Ostend, Belgium: Institute for Marine Scientific Research), 473–495.

[B57] Sorgeloos P. Lavens P. Leger P. Tackaert W. Versichele D. (1986). Manual for the Culture and Use of Brine Shrimp Artemia in Aquaculture. Belgium: State University of Ghent.

[B58] Tan J. MacRae T. H. (2018). Stress Tolerance in Diapausing Embryos of *Artemia franciscana* Is Dependent on Heat Shock Factor 1 (Hsf1). PLoS ONE 13 (7), e0200153. 10.1371/journal.pone.0200153 29979776 PMC6034868

[B59] Tanguay J. A. Reyes R. C. Clegg J. S. (2004). Habitat Diversity and Adaptation to Environmental Stress in Encysted Embryos of the crustacean *Artemia* . J. Biosci. 29 (4), 489–501. 10.1007/BF02712121 15625404

[B60] Van Stappen G. Sui L. Hoa V. N. Tamtin M. Nyonje B. Medeiros Rocha R. (2020). Review on Integrated Production of the Brine Shrimp *Artemia* in Solar Salt Ponds. Rev. Aquacult 12, 1054–1071. 10.1111/raq.12371

[B61] Vartak V. R. Joshi V. P. (2002). Effect of Different Feeds and Water Salinities on the Cyst Production of Brine Shrimp, *Artemia* Sp. J. Indian Fish. Assoc. 29, 37–47.

[B62] Versichele D. Sorgeloos P. (1980). “Controlled Production of *Artemia* Cyst in Batch Cultures,” in The Brine Shrimp Artemia. Ecology. Culturing. Use in Aquaculture. Editors Persoone G. Sorgeloos P. Roels O. Jaspers E. (Wetteren. Belgium: Universa Press), 3, 231–246.

[B63] Vos J. Léger P. Vanhaecke P. Sorgeloos P. (1984). Quality Evaluation of Brine Shrimp *Artemia* Cysts Produced in Asian Salt Ponds. Hydrobiologia 108, 17–23. 10.1007/BF0239162810.1007/bf00028178

[B64] Wang Z. Asem A. Wu J. (2019). Combined Effect of Salinity and Ferric Citrate on the Biometric Characterization of *Artemia* Cysts under Laboratory Condition. Turk. J. Fish. Aquat. Sci. 19 (5), 447–450. 10.4194/1303-2712-v19_5_09

[B65] Wang Z. C. Asem A. Sun S. C. (2017). Coupled Effects of Photoperiod, Temperature and Salinity on Diapause Induction of the Parthenogenetic *Artemia* (Crustacea: Anostraca) from Barkol Lake. China. North-Western J. Zoolog. 13 (1), 12–17. (Article No. e161302).

[B66] Wen-Ming M. Li H.-W. Dai Z.-M. Yang J.-S. Yang F. Yang W.-J. (2013). Chitin-binding Proteins of *Artemia* Diapause Cysts Participate in Formation of the Embryonic Cuticle Layer of Cyst Shells. Biochem. J. 449, 285–294. 10.1042/BJ20121259 23013449

[B67] Ye H.-L. Li D.-R. Yang J.-S. Chen D.-F. De Vos S. Vuylsteke M. (2017). Molecular Characterization and Functional Analyses of a Diapause Hormone Receptor-like Gene in Parthenogenetic *Artemia* . Peptides 90, 100–110. 10.1016/j.peptides.2017.01.008 28174072

[B68] Zhou R. Sun Y.-X. Yang W.-J. Yang F. (2014). Identification and Characterization of a *Ste20-like Kinase* in *Artemia* and its Role in the Developmental Regulation and Resistance to Environmental Stress. PLoS ONE 9 (3), e92234. 10.1371/journal.pone.0092234 24637947 PMC3956927

