# Supplementary material for: Effect of Different Culture Conditions on Gene Expression Associated With Cyst Production in Populations of Artemia franciscana
Source: Front Genet. 2022 Mar 31;13:768391. doi: 10.3389/fgene.2022.768391 (PMC9009394; doi:10.3389/fgene.2022.768391)
Supplement: Supplementary file 1 [file Table1.DOCX]

| Supplementary TABLE 1. Primers used in polymerase chain reactions. | | | |  |
| --- | --- | --- | --- | --- |
| Primer  name | GenBank  Accession  number | Sequence (5’ -3’) | Length  (pb) | References |
| *SGEG* | OM525818-  OM525823 | F: 5-CAGAGGAAGCTGCCAAAGAA-3  R: 5-TCCTTTGCCACGACTACAATC-3 | 230 | Proper design |
| *Arp-CBP* | DW678180 | F: 5-CACCAATCCCATAGAGCAT-3  R: 5-GAATGCCCGAATAGACAAC-3 | 150 | Qiu et al., 2007 |
| *BRCA1* | DW678176 | F: 5-GACCCTGGTTTATTCACACTGCT-3  R: 5-AATAATTCCGCTTCATCCACTTG-3 | 180 | Qiu et al., 2007 |
| *p8* | DW678166 | F: 5-GACGAAACAGGAAGCTGCTC-3  R: 5-TACCAGCACCATTGTCCACA-3 | 240 | Proper design |
| *Artemin* | AY062896 | F: 5-AGATGCCTTTTCCCATTGTG-3  R: 5-CTTGTGAACCGATGCAGTGT-3 | 170 | Chen et al., 2007;  King et al., 2014 |
| *ArHsp21* | DQ361273 | F: 5-TGTTCAAAGCGTTACCATCG-3  R: 5-CATCAGATGTTTTCTGCTCGC-3 | 200 | King et al., 2013;  Clegg et al., 1994;  King and MacRae, 2012 |
| *ArHsp22* | DQ361274 | F: 5-CCATGCAACTACCAGGCTTT-3  R: 5-GAAGTTCTCCATCCGACGAA-3 | 200 | King et al., 2013 |
| *p26* | DW678189 | F: 5-GCACTTAACCCATGGTACGG-3  R: 5-TCATCAGCTGTGTCCCTCAA-3 | 200 | King et al., 2014 |
| β*-Actin* | AB196465 | F: 5-AGAGCTACGAGCTGCCTGAC-3  R: 5-GTGTTGGCGTACAGGTCCTT-3 | 180 | Ojima et al., 2005 |
