# Supplementary material for: Effect of Different Culture Conditions on Gene Expression Associated With Cyst Production in Populations of Artemia franciscana
Source: Front Genet. 2022 Mar 31;13:768391. doi: 10.3389/fgene.2022.768391 (PMC9009394; doi:10.3389/fgene.2022.768391)
Supplement: Supplementary file 2 [file Table2.DOCX]

| Supplementary TABLE 2. Summary of biometrical characters for cyst under different treatments in Barro Negro (BNE) and San Francisco Bay (SFB)  populations. | | | | | | | | | | |
| --- | --- | --- | --- | --- | --- | --- | --- | --- | --- | --- |
|  | Diameter BNE (µm) | | | | | Diameter SFB (µm) | | | | |
|  | Hydrated cyst | | Decapsulated cyst | | Chorion  Thickness  (µm) | Hydrated cyst | | Decapsulated cyst | | Chorion  Thickness  (µm) |
| Treatments | No. of cysts | (±SD) | No. of cysts | (±SD) |  | No. of cysts | (± SD) | No. of cysts | (± SD) |  |
| DUN-35ppt-12L:12D-0[Fe] | 399 | 244.61^a^(15.33) | 346 | 227.31^a^(17.35) | 8.65 | 132 | 213.21^ab^ ( 8.51) | 108 | 206.88^ab^(10.92) | 3.17 |
| DUN-35ppt-12L:12D-5[Fe] | 136 | 231.64^b^(22.37) | 180 | 223.92^ab^(17.48) | 3.86 | - | - | - | - | - |
| DUN-35ppt-24L:00D-0[Fe] | 95 | 239.13^ab^(17.27) | 127 | 224.81^ab^ (17.76) | 7.16 | 131 | 224.91^c^(14.57) | 137 | 217.83^c^(16.70) | 3.68 |
| DUN-35ppt-24L:00D-5[Fe] | 103 | 230.27^b^(20.88) | 100 | 212.01^cd^ (19.04) | 9.13 | 193 | 216.37^a^( 12.01) | 160 | 202.71^ade^(14.11) | 6.83 |
| DUN-75ppt-12L:12D-0[Fe] | 69 | 219.16^c^(9.98) | 52 | 198.97^e^(12.27) | 10.09 | 349 | 207.47^d^( 11.77) | 330 | 196.82^fg^(13.04) | 5.33 |
| DUN-75ppt-12L:12D-5[Fe] | 98 | 238.2^ab^(9.83) | 69 | 217.98^bd^ (8.60) | 10.11 | 260 | 212.36^b^(10.38) | 244 | 199.06^df^(13.04) | 6.65 |
| DUN-75ppt-24L:00D-0[Fe] | 105 | 223.23^c^(7.63) | 92 | 208.25^ce^ (9.38) | 7.49 | 309 | 215.48^a^(15.44) | 308 | 200.07^de^(16.32) | 7.71 |
| DUN-75ppt-24L:00D-5[Fe] | 19 | 212.40^c^ (13.49) | 12 | 207.16^bcde^(12.00) | 2.62 | 239 | 210.70^bd^(14.59) | 228 | 192.51^g^(15.79) | 8.97 |
| TETRA-35ppt-12L:12D-0[Fe] | 28 | 237.48^ab^(11.78) | 20 | 233.44^af^(10.75) | 2.02 | 85 | 202.98^e^(7.02) | 73 | 192.48^g^(9.60) | 5.25 |
| TETRA-35ppt-12L:12D-5[Fe] | 166 | 233.45^b^ (11.94) | 175 | 213.50^cd^(12.61) | 9.97 | 141 | 235.35^f^(12.18) | 104 | 208.26^b^(12.86) | 13.54 |
| TETRA-35ppt-24L:00D-0[Fe] | 86 | 237.85^ab^(9.91) | 80 | 209.88^cd^(25.64) | 13.99 | 57 | 212.69^abd^(16.04) | 54 | 206.11^abe^(14.49) | 3.29 |
| TETRA-35ppt-24L:00D-5[Fe] | 111 | 240.90^a^ (23.52) | 85 | 236.97^f^(23.16) | 1.96 | 86 | 218.21^ac^(13.61) | 72 | 202.38^abdef^(13.81) | 7.92 |
| TETRA-75ppt-12L:12D-0[Fe] | 30 | 234.11^ab^(7.41) | 34 | 214.83^bcd^(7.92) | 9.64 | - | - | - | - | - |
| TETRA-75ppt-12L:12D-5[Fe] | - | - | - | - | - | 74 | 210.86^bd^(8.86) | 73 | 204.30^abde^(10.51) | 3.28 |
| TETRA-75ppt-24L:00D-0[Fe] | 95 | 238.76^ab^(16.84) | 76 | 230.69^af^(17.39) | 4.04 | 375 | 215.89^a^(10.42) | 332 | 203.98^abe^(11.25) | 5.96 |
| DUN | 1024 | 235.82^§^(18.32) | 978 | 220.59^§^ (18.23) | 7.61 | 1613 | **213.18**^§^(13.64) | 1515 | **200.79**^§^(16.98) | 6.20 |
| TETRA | 516 | 237.02^§^(15.83) | 470 | 220.85^§^ (20.88) | 8.08 | 818 | **217.47**^£^(14.42) | 708 | **203.46**^£^(11.96) | 7.01 |
| 35ppt | 1043 | **238.19**^§^(18.16) | 1113 | **222.53**^§^ (19.62) | 7.83 | 825 | **219.02**^§^(15.26) | 708 | **207.03**^§^(15.45) | 6.00 |
| 75ppt | 497 | **232.10**^£^(15.34) | 335 | **214.54**^£^ (15.92) | 8.78 | 1606 | **212.37**^£^(12.81) | 1515 | **199.12**^£^(14.02) | 6.63 |
| 0[Fe] | 907 | **237.80**^§^(16.28) | 772 | **222.41**^§^(19.64) | 7.70 | 1438 | **213.44**^§^(13.46) | 1342 | **202.84**^§^(14.97) | 5.30 |
| 5[Fe] | 633 | **233.95**^£^(18.96) | 676 | **218.70**^£^(18.33) | 7.62 | 993 | **216.34**^£^(14.70) | 881 | **199.82**^£^(14.74) | 8.26 |
| 12L:12D | 926 | **237.57**^§^(16.68) | 876 | 221.10^§^(17.19) | 8.24 | 1041 | **213.07**^§^(14.08) | 932 | **200.09**^§^(13.33) | 6.49 |
| 24L:00D | 614 | **234.18**^£^(18.57) | 572 | 220.04^§^(21.76) | 7.07 | 1390 | **215.79**^£^(13.92) | 1291 | **202.76**^£^(15.99) | 6.52 |
| Mean | 1540 | 236.22(17.52) | 1448 | 220.68(19.12) | 7.77 | 2431 | 214.62(14.05) | 2223 | 201.64(14.95) | 6.49 |
| Different letters among treatments indicate significant differences (Dunn test, p>0.05). Different symbols between paired groups within each factor (§ and £) indicate significant difference (bold face), according to Mann-Whitney test (p<0.05). | | | | | | | | | | |
